# Automated image-analysis method for the quantification of fiber morphometry and fiber type population in human skeletal muscle

**DOI:** 10.1186/s13395-019-0200-7

**Published:** 2019-05-27

**Authors:** Perla C. Reyes-Fernandez, Baptiste Periou, Xavier Decrouy, Fréderic Relaix, François Jérôme Authier

**Affiliations:** 1 Inserm, IMRB U955-E10, 94000 Créteil, France; 2 Faculté de Médecine, Université Paris Est Créteil, 94000 Créteil, France; 3 APHP, Hôpitaux Universitaires Henri Mondor, Centre de Référence des Maladies Neuromusculaires Nord/Est/Ile-de-France, 94000 Créteil, France; 4 Inserm, IMRB U955, Plateforme d’Imagerie, 94000 Créteil, France; 5 Etablissement Français du Sang, 94017 Créteil, France

**Keywords:** Automated image analysis, Muscle fiber quantification, Human muscle cross-sections, Fiber type populations, Biomedical imaging, Clinical diagnosis

## Abstract

**Background:**

The quantitative analysis of muscle histomorphometry has been growing in importance in both research and clinical settings. Accurate and stringent assessment of myofibers’ changes in size and number, and alterations in the proportion of oxidative (type I) and glycolytic (type II) fibers is essential for the appropriate study of aging and pathological muscle, as well as for diagnosis and follow-up of muscle diseases. Manual and semi-automated methods to assess muscle morphometry in sections are time-consuming, limited to a small field of analysis, and susceptible to bias, while most automated methods have been only tested in rodent muscle.

**Methods:**

We developed a new macro script for Fiji-ImageJ to automatically assess human fiber morphometry in digital images of the entire muscle. We tested the functionality of our method in deltoid muscle biopsies from a heterogeneous population of subjects with histologically normal muscle (male, female, old, young, lean, obese) and patients with dermatomyositis, necrotizing autoimmune myopathy, and anti-synthetase syndrome myopathy.

**Results:**

Our macro is fully automated, requires no user intervention, and demonstrated improved fiber segmentation by running a series of image pre-processing steps before the analysis. Likewise, our tool showed high accuracy, as compared with manual methods, for identifying the total number of fibers (*r* = 0.97, *p* < 0.001), fiber I and fiber II proportion (*r* = 0.92, *p* < 0.001), and minor diameter (*r* = 0.86, *p* < 0.001) while conducting analysis in ~ 5 min/sample. The performance of the macro analysis was maintained in pectoral and deltoid samples from subjects of different age, gender, body weight, and muscle status. The output of the analyses includes excel files with the quantification of fibers’ morphometry and color-coded maps based on the fiber’s size, which proved to be an advantageous feature for the fast and easy visual identification of location-specific atrophy and a potential tool for medical diagnosis.

**Conclusion:**

Our macro is reliable and suitable for the study of human skeletal muscle for research and for diagnosis in clinical settings providing reproducible and consistent analysis when the time is of the utmost importance.

**Electronic supplementary material:**

The online version of this article (10.1186/s13395-019-0200-7) contains supplementary material, which is available to authorized users.

## Background

Muscle histology analyses are very effective for the characterization of morphological parameters to determine muscular health status and an important tool for the diagnosis of numerous muscle diseases [1]. For decades, myopathological diagnosis remained based on purely qualitative evaluations, notably depending on pathologists’ experience. In the last few years, the growing development of innovative treatments, including biotherapies, requires a more stringent stratification of patients, with accurate quantification of myopathological processes. Physiological aging and pathological muscle atrophies are characterized by a reduction of myofibers’ size and number, increased myofibers’ size variation [2], and alterations in type I (slow twitch)/type II (fast twitch) myofiber ratio [3, 4]. The conventional procedure for quantifying muscle atrophy in fixed or frozen tissue consists of manual quantification of the myofibers’ size (fiber diameter at its narrowest point) to evaluate muscle morphology. Because of the large number of myofibers within a muscle section, morphology evaluation by manual methods is usually limited to a small representative area (~ 400 fibers) [5]. However, this procedure is time-consuming, labor intensive, and subjected to user bias and may not adequately represent the whole tissue complexity and/or heterogeneity. An increasing amount of software for the automated analysis of muscle images has been recently developed, yet some of these require extensive human intervention [6], are not publicly available [7], or have been only tested in rodents [8, 9] or are not designed for the analysis of human muscle in medical settings. Here, we provide a reproducible and accurate automatic image analysis macro script that runs in an open-access platform (Fiji-ImageJ) and allows for the fast quantification and differentiation of human muscle fiber composition and histomorphometry in different physiological and pathological conditions. Our macro was designed to assess fiber morphometry in digital images of the entire muscle. We tested the functionality of our method in deltoid muscle biopsies from a heterogeneous population of patients with histologically normal muscle (male, female, old, young, lean, obese), patients with pathological muscle, and a small set of pectoral samples. Thus, our method provides a potentially powerful tool for research in the skeletal muscle field and medical diagnostics.

## Methods

### Patients

We analyzed deltoid samples from patients who underwent a muscle biopsy for diagnostic purposes at the Centre for Neuromuscular Disorders of the Henri Mondor University Hospital. Fifty-seven patients with histologically normal muscle (27 males, 30 females; age 16–87 years) were included in the study. Subjects were divided into four age (15–29, 30–49, 50–69, and > 70 years old) and four body mass index (BMI) (17–18.4, 18.5–24.9, 25–29.9, > 30 kg/m^2^) groups. Subjects’ characteristics are summarized in Table 1. For the validation of our tool in pathological conditions, we obtained deltoid samples from dermatomyositis (DM, 4 males, 4 females; age 17–84), necrotizing autoimmune myopathy (NAM, 4 females; age 23–62), and anti-synthetase myopathy (ASM, 2 males, 2 females, age 49–82) patients. Diagnosis of DM, NAM, and ASM was established by physicians from the Centre for Neuromuscular Disorders of the Henri Mondor University Hospital based on the combination of clinical, biological, electromyogram, and histopathological features following the European NeuroMuscular Centre (ENMC) criteria [10]. For the diagnosis of ASM, the presence of circulating anti-synthetase auto-antibodies was required. In addition, we evaluated two muscle samples with neurogenic atrophy or unspecific myofiber atrophy [11]. Opportunely, a project in our laboratory exploring pectoral muscle morphometry in patients undergoing cardiac surgery and currently on the specimen collection stages allows us access to some of these biopsies. Thus, we used a set of ten pectoral muscle samples (nine males, one female; age 29–65 years; BMI 21.9–33.5 kg/m^2^) to test our macro performance in a muscle type other than deltoid. The study was conducted in accordance with the French legislation on muscle samples from the Henri Mondor Hospital Biological Resource Platform (registration number in the French Ministry of Health #DC-2009-930; authorization for research # AC-2014-2056). All patients gave written consent for their participation.

**Table 1:** Subjects’ characteristics

	Males (*n* = 27)			Females (*n* = 30)		
	Mean ± SEM	Max	Min	Mean ± SEM	Max	Min
Age (years)	49.4 ± 3.7	87	16	48.2 ± 3.2	83	21
Height (m)	1.75 ± 0.01	1.86	1.62	1.64 ± 0.01	1.77	1.48
BW (kg)	74.3 ± 2.83	109	53	66.8 ± 3.8	120	40
BMI (kg/m^2^)	24.1 ± 0.76	32.2	18.1	24.7 ± 1.3	42.5	17.2

Values are means ± SEM (*n* = 57). *BW* body weight, *BMI* body mass index

### Preparation of histological specimens

Open biopsies were taken from the deltoid muscle under local anesthesia. Pectoralis major muscle biopsies (∼ 1 × 2 × 1 cm) were obtained from individuals undergoing mitral or aortic valve surgery at the Henri Mondor Hospital in Creteil, France. Both deltoid and pectoral muscle biopsies were carefully collected to avoid the inclusion of tendon, or intermuscular septa or epimysial components of extracellular matrix. Muscle samples were placed in a gauze lightly dampened with saline and conventionally processed for myopathology analysis. Briefly, muscles were mounted vertically, maintaining the orientation of the fibers, in a flat piece of cork and held with a 1:1 mix of tragacanth and water. Samples were flash-frozen in isopentane cooled with liquid nitrogen and kept at − 80 °C until use. Frozen muscle samples were cut in 7 μm sections using a cryostat (CryoStar NX70, Thermo Fisher Scientific, Waltham, MA, USA) with the inner chamber temperature set at − 20 °C. The cuts were laid on SuperFrost® Plus glass slides (Thermo Fisher Scientific, Waltham, MA, USA), left at room temperature for 1 h to dry and fix, and then stored at − 80 °C until use.

### Immunofluorescence staining of muscle samples

Muscle sections were dried at room temperature for 20 min, and a working area was delimited using a DakoPen (Cat # S200230-2, Agilent, Dako). Samples were hydrated with 1X PBS for 10 min and permeabilized with 0.5% Triton for 5 min. The Endogenous Avidin/Biotin Blocking Kit (Cat # 00-4303, Invitrogen, Life Technologies) was used to reduce background signal according to the manufacturer’s instructions. Samples were rinsed with 1X PBS and incubated with 10% BSA (Sigma-Aldrich) for 30 min at room temperature. Overnight incubation at 4 °C was conducted with the primary antibodies: dystrophin rabbit polyclonal antibody (1:200) (Cat # RB-9024P, Thermo Scientific) and α-II spectrin rabbit polyclonal antibody (1:400), to target the cell membrane and the mouse monoclonal antibody for myosin heavy chain (MyHC, 1:400) (Cat # NCL-MHCf, Novocastra, Leica Biosystems) to target type II myofibers. The next day, the samples were washed with 1X PBS and incubated with the secondary antibodies (1:500): goat anti-rabbit FITC 488 (Cat #A11034, Thermo Fisher Scientific) and biotinylated goat anti-mouse IgG (Cat # BA-9200, VectorLabs) for 30 min at 37 °C for membrane and fast myosin, respectively. Samples were rinsed in 1X PBS and incubated with Dylight 549 Streptavidine Cy3 (1:500) (Cat # SA-5549, VectorLabs) for 30 min at 37 °C. Samples were rinsed again, mounted, and stored protected from light at 4 °C until visualization. All samples analyzed were processed by block randomization, and investigators assessing the experimental outcomes were blinded to age, gender, and body mass index (BMI) groups.

### Image acquisition and image format for the macro

Slides were scanned and digitized using an Axio Imager D1 Zeiss microscope. Once the sample was in place, the focus and exposure were adjusted to improve the sharpness of the image and light intensity. The camera used for image acquisition has a dynamic range of 0–4095 (0 = no intensity, 4095 = the highest pixel intensity value). Based on previous experiences, the ideal exposure time to acquire the images was selected when the intensity of the signal was ~ 80–85% of the dynamic range (i.e., histogram intensity ~ 3200–3500). Whole muscle sections were captured with a × 10 objective in mosaic, using the tiles option. Once the pictures of the individual channels (FICT 488 (green) for the membrane and Cy549 (red) for the MyHC) were acquired, stitch post-processing was applied to merge the pictures, correct alignments, and relate the pixel coordinates of one image to the other. Individual channel- and two channel-merged images were saved in a TIF format without compression. Each merged image had a size of approximately 9300 × 9900 pixels and ~ 200 Mb. Original pictures of each channel (16 bit) were used for the automated analysis.

### Automated morphometric analyses using our macro in Fiji

Morphometric analyses were conducted using Fiji (Fiji is just ImageJ), an open-source image processing package based on ImageJ® [12], in the digitized images of the entire muscle section. To automatically detect and quantify the myofibres in the sections, we generated a customized macro script for the assessment of human histological samples in collaboration with the IMRB image platform (Creteil, France).

### Overview of the developed macro

**Fig. 1:**
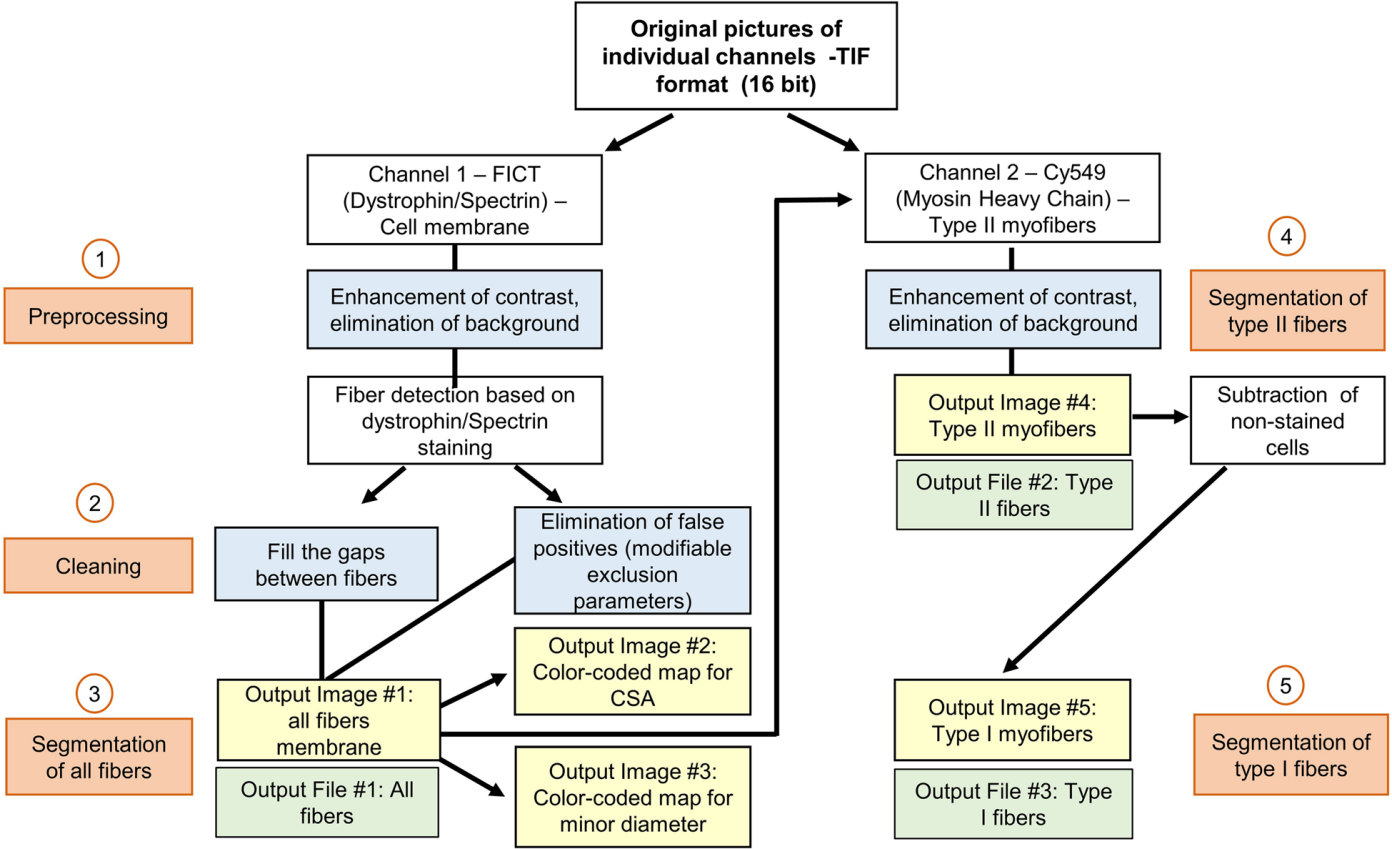
Automated analysis pipeline using our macro using Fiji. Automated analysis of immunofluorescence-stained muscle sections using our developed macro in the Fiji platform. (1) The first step consists of image enhancement by eliminating background noise and increasing the contrast. (2) The macro includes a line of command to fill the gaps within the fibers and a list of user-modifiable exclusion parameters to improve fiber detection and reduction of artifacts. (3) Then, individual fibers’ borders are identified based on the dystrophin/spectrin staining. (4) Among the detected fibers, those positive for MyHC are identified and segmented as type II myofibers. (5) Unlabeled type I myofibers are then calculated by difference. Fibers’ morphology parameters are computed, and the results automatically saved as .xls files. The program automatically saves the segmentation results of all and each fiber type as a flattened image, as well as the color maps for the area and minor diameter size distribution

The macro is entirely automated and requires no user intervention or clicking in dialog boxes during analysis. The macro can be run in Fiji in a computer with available Java 8 runtime. The image processing is compatible with Windows 64-bit (XP, Vista, 7, 8, and 10), Mac OS (X 10.8), Mac OS X, and Linux (× 64 and × 86 architectures). The pipeline of the macro instructions is depicted in Fig. 1. After selection of a folder containing the individual channel images (one for the membrane: C1; one for type II fibers: C2) of each sample, the program will automatically load the files within the selected folder to conduct the analysis.

**Fig. 2:**
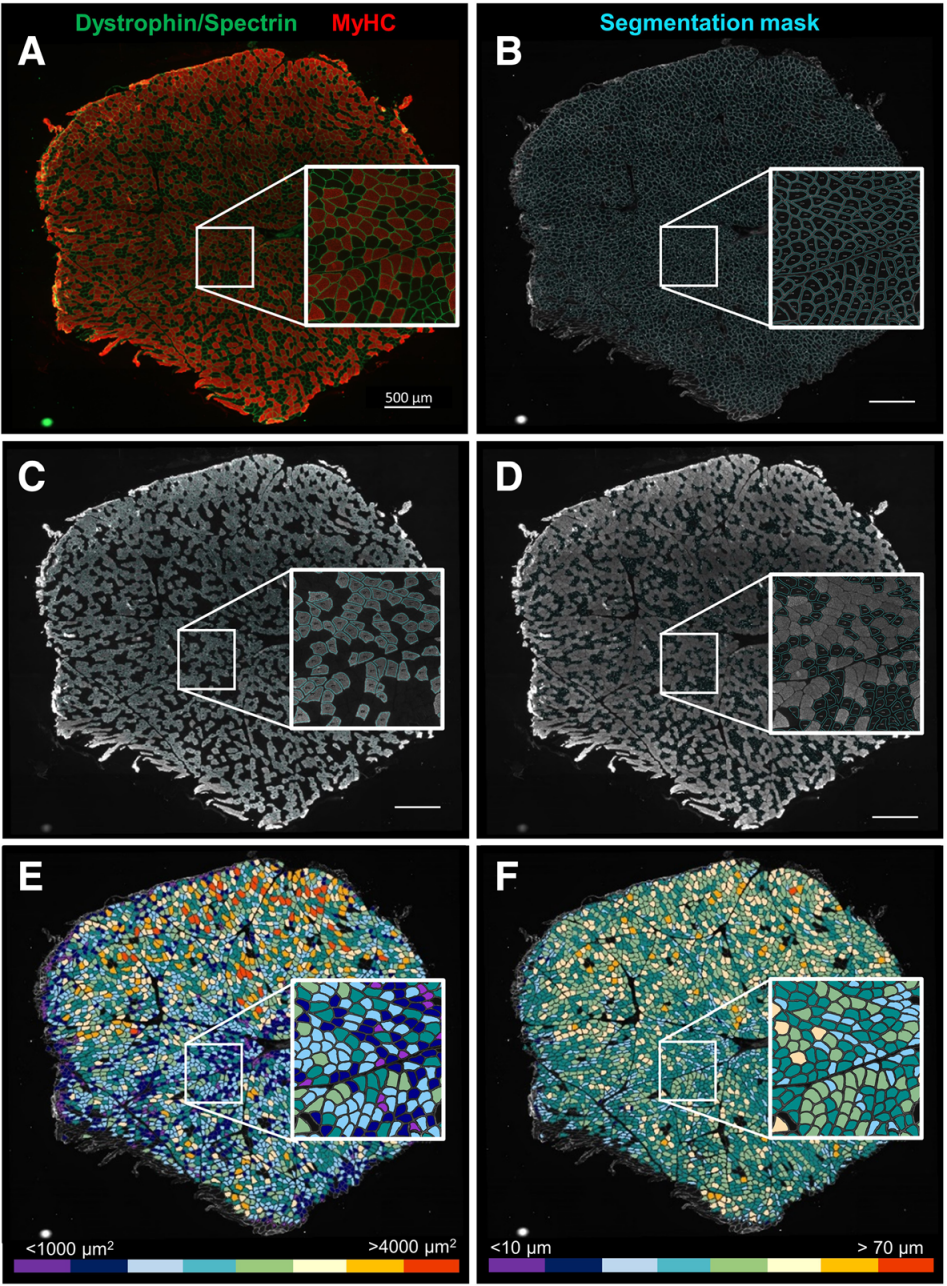
Example of automated analysis results. Human deltoid muscle cross-section analyzed with our macro in Fiji-ImageJ. **a** Cross-sections are labeled for dystrophin and spectrin (green—channel 1) to outline the fibers and for myosin heavy chain (MyHC) (red—channel 2) to identify type II myofibers; type I myofibers are unlabeled. Images were acquired with a Zeiss epifluorescence microscope using a × 10 objective and saved in a 16-bit TIF format. Original pictures of individual channels were used for the analysis. **b** After image enhancement and cleaning steps, channel 1 is used to segment and outline the membrane of all fibers; a flattened image is automatically saved. **c** Channel 2 is then used to identify labeled cells within those previously identified cells. **d** Non-labeled fibers are then calculated by difference, and type I myofibers are segmented. To facilitate the visual identification of fiber’s size distribution, color-coded maps were obtained based on the myofibers. **e** Cross-sectional area (from ≤ 1000 to > 4000 μm^2^). **f** Minor diameter (from ≤ 10 to > 70 μm). Scale bar represents 500 μm

Image processing is conducted using mathematical morphology. The first steps consist of automatic image enhancement by (1) eliminating background noise (despeckle filter), (2) increasing the contrast of the image, and (3) subtracting the background (clear out). Then, individual fibers’ borders are identified based on the dystrophin/spectrin staining of the sarcolemma (C1—green). The macro includes a line of command to fill the gaps within the fibers and a list of user-modifiable exclusion parameters to improve fiber detection and reduction of artifacts. These exclusion parameters can be modified in a pop-up window that appears before the analysis. In the analyses presented here, the following parameters were excluded: CSA < 200 and > 13,000 μm^2^, circularity < 0.4, and minor diameter < 1.5 μm. After the cleaning steps, the program automatically saves the segmentation results as a flatten image (Fig. 2b). These results are used for the following steps. Among the detected fibers, those positive for MyHC (C2—red) are identified and segmented as type II myofibers (Fig. 2c). Unlabeled type I myofibers were then calculated by difference (Fig. 2d).

Cross-sectional area (CSA) was determined using a pixel to micrometer conversion factor estimated with a precision ruler (0.647 μm/pixel). CSA (μm^2^), perimeter (μm), major (μm) and minor diameters (μm), and circularity are computed for all myofibers, and the results obtained from these analyses are automatically saved as Excel (.xls) files for all, type I, and type II myofibers separately. Finally, to facilitate visual analysis of fiber size distribution, a color map for each section was created based on the myofibers’ size. From smaller to larger, cells were color-coded as follows for area (Fig. 2E) and minor diameter (Fig. 2F), respectively: dark orchid, ≤ 1000 μm^2^ and ≤ 10 μm; night blue, 1000–1500 μm^2^ and 11–20 μm; cyan blue, 1501–2000 μm^2^ and 21–30 μm; dark cyan, 2001–2500 μm^2^ and 31–40 μm; dark sea green, 2501–3000 μm^2^ and 41–50 μm; yellow, 3001–3500 μm^2^ and 51–60 μm; orange, 3501–4000 μm^2^ and 61–70 μm; and red, > 4000 μm^2^ and > 70 μm. All output results (images and .xls files), as well as a log file for each sample with information of the total area of tissue detected (μm^2^) (i.e., calculated surface of analysis) and number of total, type I, and type II fibers, will be saved in the same folder selected for analysis. The program runs in a loop, and once analyses are completed, a pop-up message in the computer screen will appear to inform the user.

### Validation of macro’s performance

To validate the sensitivity and accuracy of our macro compared to manual analysis, we conducted a test in a small section (~ 300 myofibers) of a subset of random samples from healthy patients (*n* = 10–15). The number of total, type I, and type II fibers was counted, and the minor diameter of the fibers was measured either manually (using the ruler tool of Fiji-ImageJ) or using our developed macro (automated analysis). The macro’s utility in clinical diagnostics was determined in muscle samples from a wide-ranging cohort of healthy patients and in a subset of patients with muscle disease, diagnosed based on the European NeuroMuscular Centre (ENMC) criteria [10]. The quality and accuracy of the output images obtained from all our analysis (single fiber segmentation and fiber type identification) were visually assessed.

### Statistical analysis

The total number of fibers per sample was normalized by dividing this value by the tissue area (computed by the macro in μm^2^) and then multiplying this result by 10^7^, hence obtaining the number of fibers in a 10-mm^2^ area. For each sample, the averages for CSA, perimeter, and major and minor diameters were computed and used for all, type I, and type II fiber analyses. Because sex-related differences were found to affect muscle architecture in our population (Additional file 1: Table S1), we removed the confounding effects of gender on fiber size and number by conducting a gender-adjusted two-way analysis of covariance (ANCOVA). The main effects of age, BMI, and their interactions were assessed for all, type I, and type II fibers. The data are presented as least square means with a standard error of the mean (LSMeans ± SEM). Tukey-Kramer post hoc test was used to determine differences between groups. Pearson’s correlation coefficients were conducted to establish significant associations among the parameters of interest. Statistical analyses were performed using SAS Enterprise Guide OnDemand (SAS Institute, Inc., Cary, NC), and significance was set at *p* < 0.05. For the analysis of pathological and pectoral muscle samples, we conducted basic summary statistics, and to quantify the degree to which fibers were abnormally small or large in a biopsy, we calculated atrophy and hypertrophy factors based on the fiber size distribution. A score was assigned to each of the fibers in a sample according to its CSA (for atrophy, CSA ≤ 500μm^2^, score 20; ≥ 501 to ≤ 1000, 10; ≥ 1001 to ≤ 1500, 5; ≥ 1501 to ≤ 2000, 1; and ≥ 2001, 0; and for hypertrophy, CSA ≥ 4501 to ≤ 5500 μm^2^, score 1; ≥ 5501 to ≤ 6500, 5; ≥ 6501 to ≤ 7500,10; ≥ 7501 to ≤ 8500, 20; and ≥ 8501, 30). The sum of these scores was divided by the number of fibers (all, type I, or II) within a section. Hence, the higher the score for each factor, the greater the presence of abnormal fiber size.

## Results

### Immunofluorescence staining and automated morphometric analyses using Fiji

One of the challenges of conducting automated analyses using immunofluorescence-stained samples is that high fluorescence signals are required and the myofibers membrane need to be completely closed to be properly identified. Moreover, some images need to be pre-treated to increase brightness and contrast using different image editing software before analysis, which can be very time-consuming. We overcame these problems in two steps: First, the immunostaining protocol used was carefully optimized in muscle frozen sections (i) to increase the fluorescence signal without increasing the background and (ii) to ensure closure of the membrane, and its following identification by our automated program, by combining two different antibodies to target the sarcolemma. Second, the macro also includes instructions to enhance the image automatically, eliminate artifacts, and fill small gaps within a fiber before analysis, thus improving the detection of individual fibers. Our macro works optimally in images acquired with a broad range of exposure (from 30 to 170% of the ideal time exposure (see the “Methods” section)). However, its performance will be compromised, and detection gradually lost when analyzing images acquired below 20% (low signal) or above 300% (very high signal) of our reference exposure time.

### Macro’s performance and validation

The average time for the automated analysis of a whole deltoid muscle section (~ 3000 fibers) for the segmentation and identification of fiber type populations using our macro is ~ 5 min. The duration of the analysis is greatly diminished in comparison with manual (up to 1 h/selected field of ~ 400 myofibers), semi-automatic (~ 15 min [6]), or automated methods that require image editing before analysis. Compared with the manual analysis (*n* = 15), our macro showed high accuracy for identifying the total number of fibers (*r* = 0.97, *p* < 0.001), the fiber I and fiber II proportion (*r* = 0.92, *p* < 0.001), and minor diameter (*r* = 0.86, *p* < 0.001) (Additional file 1: Figure S1). The variability observed between the macro and manual approaches for measuring minor diameter could be mostly due to human error, given the imprecision in segmentation, arbitrary selection of measure location, and user bias to which manual analyses are usually subjected to.

Similarly, the output of the analyses was considered highly satisfactory when visually comparing the digitized muscle images with the flattened, segmented masks for all fibers and each fiber type. The macro analysis resulted in an overall detection/segmentation of 89.3% of the total fibers (342/3212 not detected fibers across 10 samples analyzed). However, within the detected fibers, our tool showed a very high rate of accuracy for determining fiber type populations, with < 1% of the fibers being wrongly categorized (21/3212). An essential aspect of this automated method is that the morphometry evaluation is no longer subjected to user-bias. Thus, inter-user variability is greatly decreased, and the program ensures consisting and reproducible results across analyzed samples.

### Analysis of human skeletal muscle samples using our macro tool in Fiji

#### The proportion of type-specific fibers does not change with age or BMI

The macro was tested in 57 deltoid samples of subjects with histological normal muscle across different age (15–29 (very young), 30–49 (young), 50–69 (middle age), and > 70 (old) years old) and BMI groups (17–18.4 (underweight), 18.5–24.9 (normal weight), 25–29.9 (overweight), and > 30 kg/m^2^ (obese)). We analyzed CSA, perimeter, and major and minor diameters of all, type I, and type II myofibers, separately. Because biopsy sizes were heterogeneous in our population (mean 13.5 ± 0.6 mm^2^, range 3.6 to 24.9 mm^2^), we normalized the total number of fibers by tissue area. The average number of fibers (per 10 mm^2^ area) in a deltoid section was 2283.47 ± 109.5 (Additional file 1: Table S2), and in our population, this value was not different across age (*p* = 0.72) groups. We observed a trend for a significant BMI main effect (*p* = 0.07) towards a smaller number of fibers/area in the obese vs. lean groups (Additional file 1: Table S3). We also computed the percentage of type I and type II myofibers present in each sample based on the total number of fibers quantified. The average proportion of myofiber population in adult deltoid muscles was 47.4 ± 1.7 and 52.6 ± 1.7% for type I and type II, respectively (Additional file 1: Table S2). In our population, age did not significantly affect the percentage distribution of type I or type II (*p* = 0.47) myofibers (Additional file 1: Table S3). In the youngest group (15–29 years old), the distribution of type I/II population was 39.9/60.1%, while in the oldest group (70–89 years old) was 50.1/49.9%, respectively (Table 2). We did not observe differences in fiber population distribution across BMI groups either (*p* = 0.98) (Additional file 1: Table S3). Underweight individuals exhibited a 48.4/51.6% of type I/II fibers, compared to a 45.9/54.1% found in obese subjects (Additional file 1: Table S4).

**Table 2:** Comparison of mean myofibers’ morphology across age groups

	Age group (years old)			
Myofibers’ morphology	15–29	30–49	50–69	70–89
Number of fibers (by area)^±^	2225.7 ± 330.4	2238.9 ± 190.5	2567.6 ± 252.3	2235.5 ± 262.1
Number of type I (%)	39.9 ± 5.7	46.7 ± 3.3	50.4 ± 4.4	50.1 ± 4.5
Number of type II (%)	60.1 ± 5.7	53.3 ± 3.3	49.6 ± 4.4	49.9 ± 4.5
CSA all fibers (μm^2^)	2362.3 ± 253.6^ab^	2568.8 ± 146.3^b^	2534.8 ± 193.7^ab^	1868.6 ± 201.2^a^
CSA type I (μm^2^)	2397.5 ± 263.1	2371.6 ± 151.8	2279.7 ± 201	2236.8 ± 208.8
CSA type II (μm^2^)	2359.5 ± 313^ab^	2771.9 ± 180.5^b^	2654.0 ± 239.1^b^	1443.2 ± 248.4^a^
Perimeter all fibers (μm)	205.7 ± 11.7^ab^	214.1 ± 6.7^b^	210.3 ± 8.9 ^ab^	179.0 ± 9.3^a^
Perimeter type I (μm)	205.0 ± 11.4	204.0 ± 6.5	199.4 ± 8.7	198.8 ± 9.0
Perimeter type II (μm)	207.2 ± 14.9^ab^	224.2 ± 8.6^b^	215.4 ± 11.4^b^	160.4 ± 11.8^a^
Major diameter all fibers (μm)	70.1 ± 3.8^ab^	73.1 ± 2.2^b^	71.8 ± 2.9^ab^	61.0 ± 3.1^a^
Major diameter type I (μm)	69.9 ± 3.7	69.8 ± 2.1	68.0 ± 2.8	66.8 ± 2.9
Major diameter type II (μm)	70.6 ± 4.9^ab^	76.4 ± 2.8^b^	73.5 ± 3.7^b^	55.4 ± 3.9^a^
Minor diameter all fibers (μm)	41.2 ± 2.1^b^	43.1 ± 1.2^b^	41.8 ± 1.6^b^	33.3 ± 1.7^a^
Minor diameter type I (μm)	42.6 ± 2.2	42.1 ± 1.3	41.0 ± 1.7	38.1 ± 1.8
Minor diameter type II (μm)	40.5 ± 2.6^b^	44.1 ± 1.5^b^	41.4 ± 2.0^b^	28.6 ± 2.0^a^

Morphology of total, type I, and type II myofibers of deltoid muscle samples evaluated using our macro run in Fiji-ImageJ. Analysis of covariance (two-way ANCOVA) results by age group are shown. Averages of each sample were used for all, type I, and II fiber analysis. Values are LSmeans ± SEM. For each parameter, means in the same row that have no superscript in common are significantly different from each other (Tukey-Kramer test, *p* < 0.05). *CSA* cross-sectional area. ±Normalized number of fibers/area (10 mm^2^)

#### Age results in size decrease of type II but not type I myofibers

The average cross-sectional area for all fibers was 2399.4 ± 106.5 μm^2^, their average perimeter 206.6 ± 5 μm, and their major and minor diameters 70.5 ± 1.7 and 40.9 ± 1 μm, respectively (Additional file 1: Table S2). Overall, all fibers’ CSA (*p* = 0.045), perimeter (*p* = 0.03), and major (*p* = 0.02) and minor (*p* = 0.0003) diameters were negatively affected by an age main effect (Additional file 1: Table S3). While there were no differences in the all fibers’ morphometry parameters among the 15–29, 30–49, and 50–69 age groups, we found that ≥ 70-year-old subjects had significantly smaller CSA (− 27.3%, *p* = 0.036), perimeter (− 16.4%, *p* = 0.02), and major diameter (− 16.6%, *p* = 0.013) compared with the 30–49 (young) group. Likewise, in older individuals, the fibers’ minor diameter was ~ 20% smaller than in the other age groups (Table 2).

**Fig. 3:**
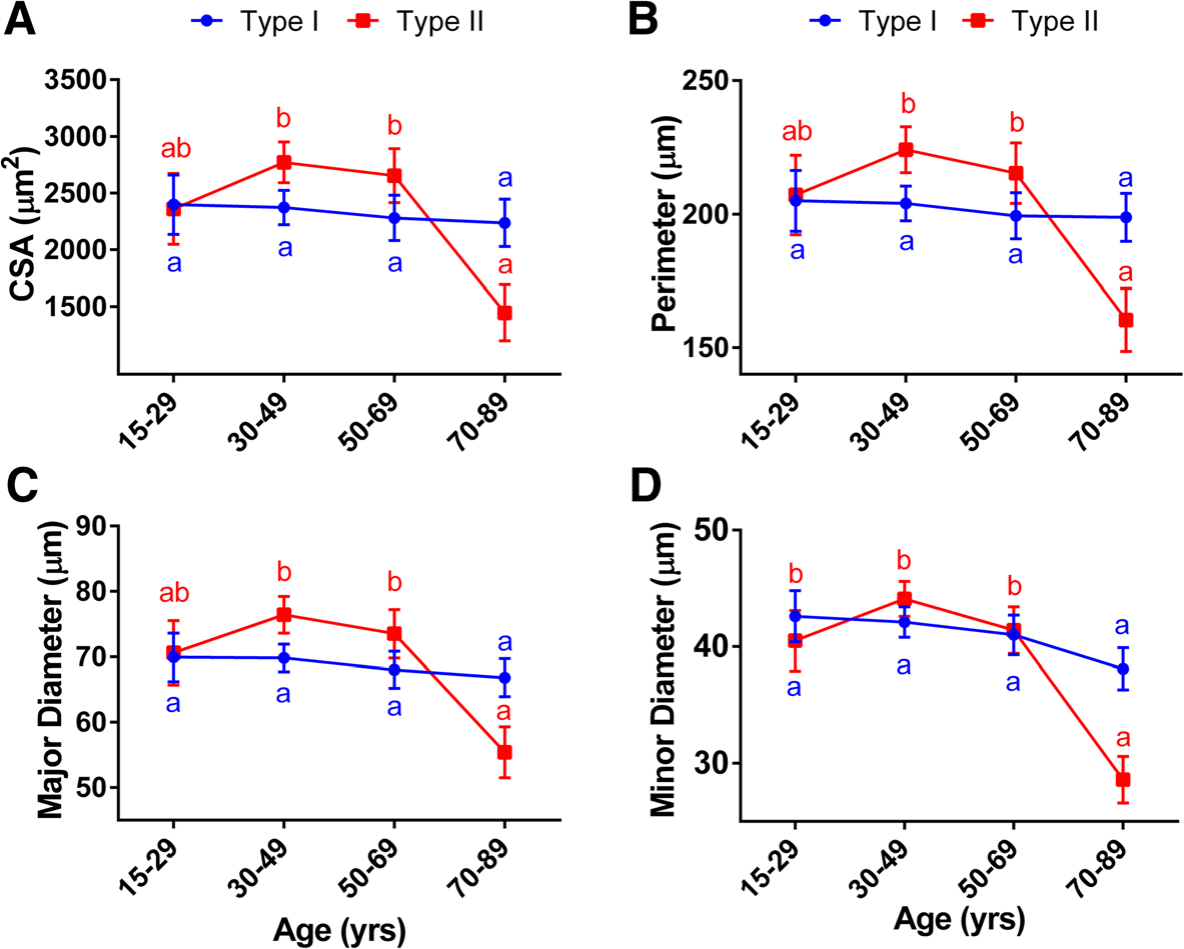
Type-specific fiber changes in morphometry by age. Deltoid muscle analysis of type-specific fiber morphometric parameters across different age groups. Each point reflects the LSmean ± SEM (*n* = 57). Averages of each sample were used for the analysis of covariance (two-way ANCOVA) of **a** cross-sectional area, **b** perimeter, and **c** major and **d** minor diameters which were analyzed. Within a fiber population, means with that have no superscript in common are statistically different across age groups (Tukey-Kramer test, *p* < 0.05)

When looking at the fiber population analysis, we found that the effects of aging were fiber type specific. Age had no effect on the morphometry of type I (CSA, *p* = 0.94; perimeter, *p* = 0.94; major diameter, *p* = 0.84; and minor diameter, *p* = 0.28), whereas the size of the type II myofibers (CSA, *p* = 0.0008; perimeter, *p* = 0.0009; major diameter, *p* = 0.0008; and minor diameter, *p* < 0.0001) decreased with aging (Fig. 3, Additional file 1: Table S3).

Similar to our observations in the all-fiber analysis, there were no significant differences in the type II fiber size among the groups of individuals younger than 70 years old. Yet, in the older age group (> 70 years), we found size reductions of 46–48% in CSA, 26–29% in perimeter, and 25–28% in major and 31–35% in minor diameters, compared with individuals in the 50–69 and 30–49 age groups, respectively (Fig. 3, Table 2).

#### Changes in the fiber type size associated with obesity

We observed significant BMI-by-age interactions for all fibers (CSA, *p* = 0.007; perimeter, *p* = 0.005; major diameter, *p* = 0.002; minor diameter, *p* = 0.002) and type II morphometry parameters (CSA, *p* = 0.017; perimeter, *p* = 0.017; major diameter, *p* = 0.008; minor diameter, *p* = 0.004), and for the major diameter of type I myofibers (*p* = 0.03). We also found a trend for an interaction affecting the perimeter (*p* = 0.08) and minor diameter (*p* = 0.05) of type I fibers. However, BMI alone did not affect the morphometry parameters evaluated for either all fibers or the specific fiber populations (Additional file 1: Table S3).

**Fig. 4:**
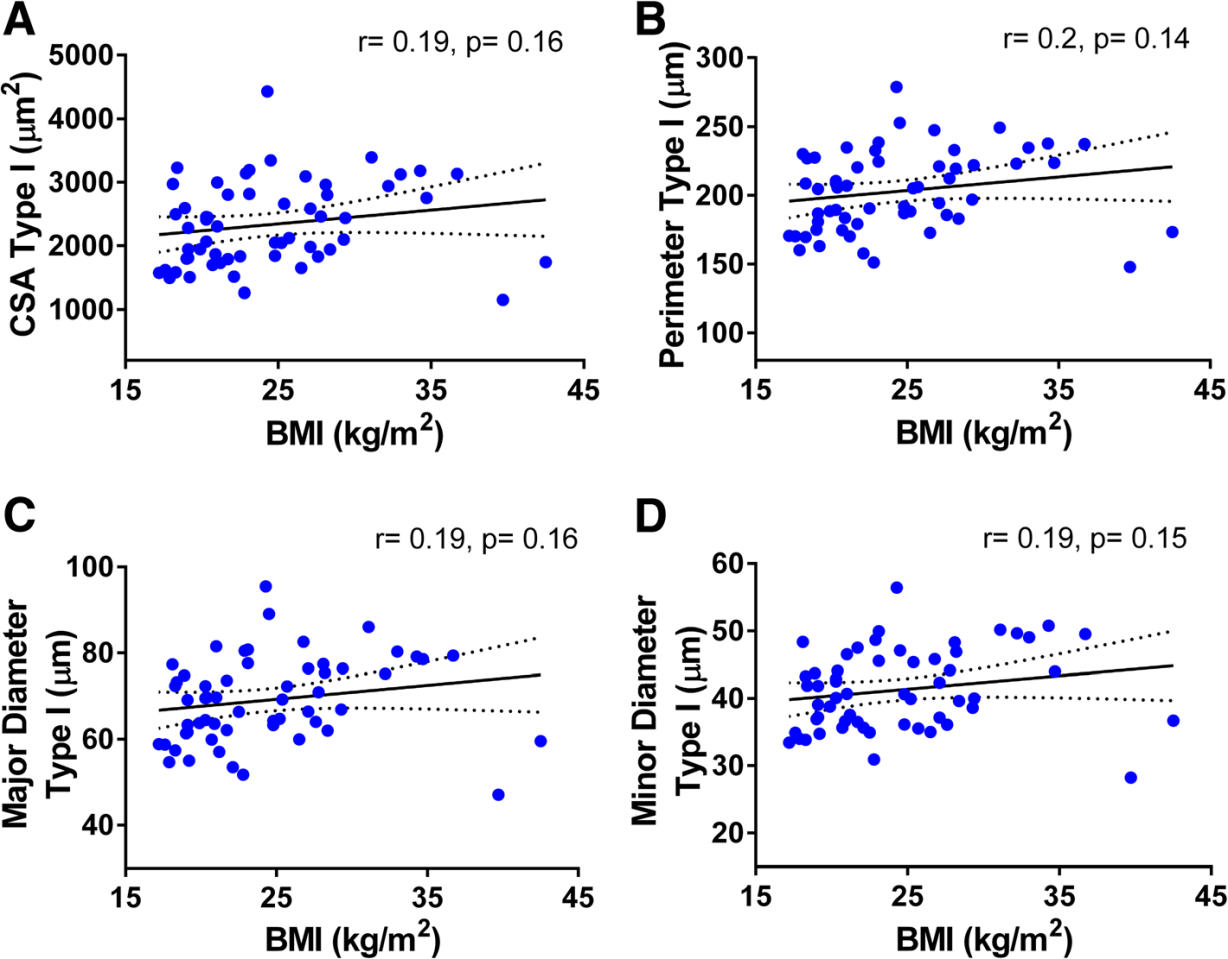
Correlations between type I-specific myofibers morphometry and BMI. Pearson’s correlation coefficient (*r*) and *p* values are shown for **a** cross-sectional area, **b** perimeter, and **c** major and **d** minor diameters of type I myofibers of deltoid muscle plotted against BMI. Averages of each subject for each parameter with 95% confidence intervals are shown (*n* = 57)

**Fig. 5:**
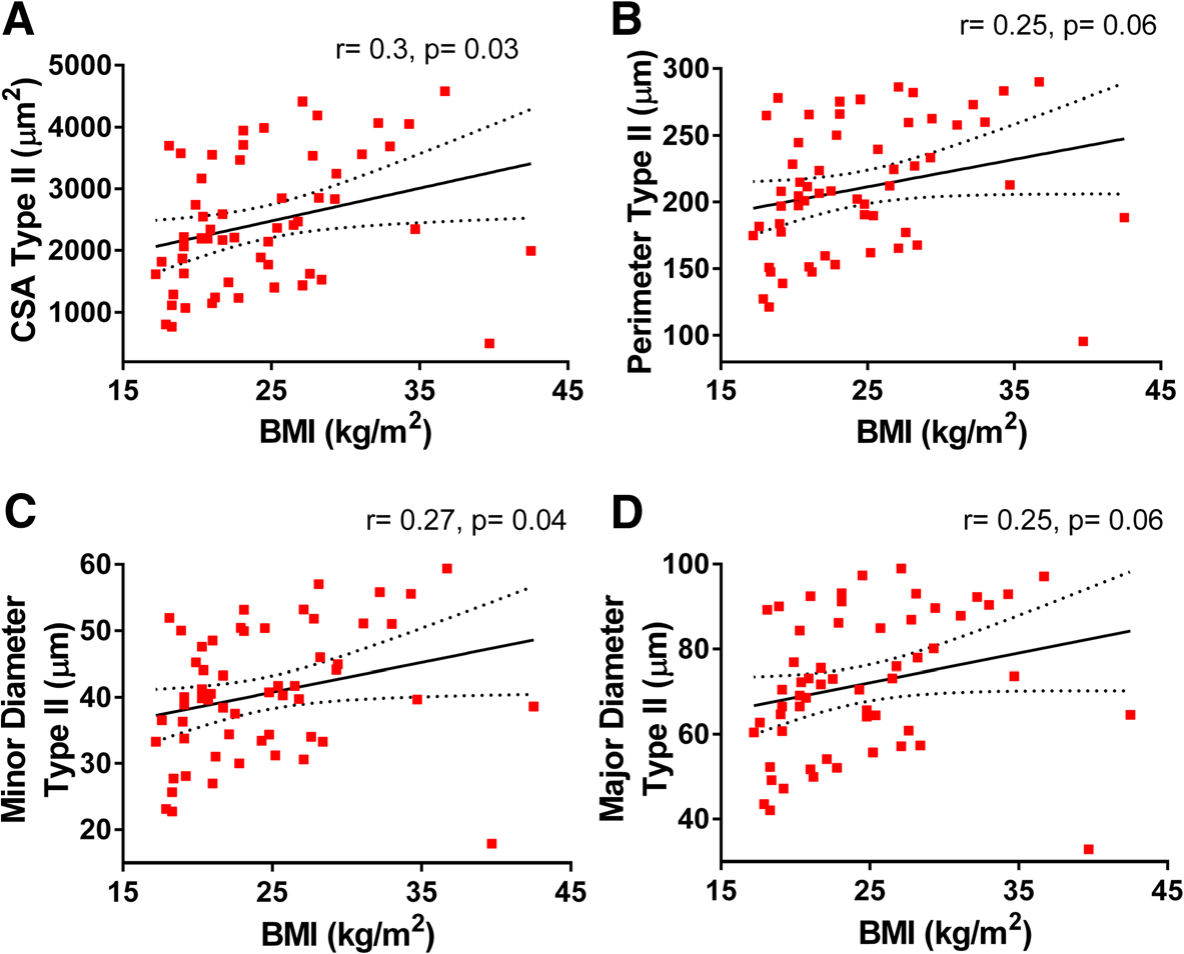
Correlations between type II-specific myofibers morphometry and BMI. Pearson’s correlation coefficient (*r*) and *p* values are shown for **a** cross-sectional area, **b** perimeter, and **c** major and **d** minor diameters of type II myofibers of deltoid muscle plotted against BMI. Averages of each subject for each parameter with 95% confidence intervals are shown (*n* = 57)

We analyzed the relationships among morphometry parameters and BMI, and while no significant correlations between BMI and type I fiber morphometry were found (Fig. 4), this was not the case for the type II population. Type II fibers’ CSA (*r* = 0.3, *p* = 0.03) and minor diameter (*r* = 0.27, *p* = 0.04) were positively and significantly correlated with BMI, while a trend for a positive association was observed for their perimeter (*r* = 0.25, *p* = 0.06) and major diameter (*r* = 0.25, *p* = 0.06) (Fig. 5). Similar associations between fiber size and BMI were observed in the whole fiber population (i.e., significant CSA (*r* = 0.28, *p* = 0.032) and minor diameter (*r* = 0.27, *p* = 0.039) and a trend for significance for perimeter (*r* = 0.25, *p* = 0.057) and minor diameter (*r* = 0.25, *p* = 0.07)). Overall, these data are consistent with the observation that subjects with greater BMI tend to have fewer fibers/area compared to leaner individuals (BMI > 30: 1627.6 ± 316.7 vs. BMI < 18.5: 2793.8 ± 313.2, Additional file 1: Table S4).

**Fig. 6:**
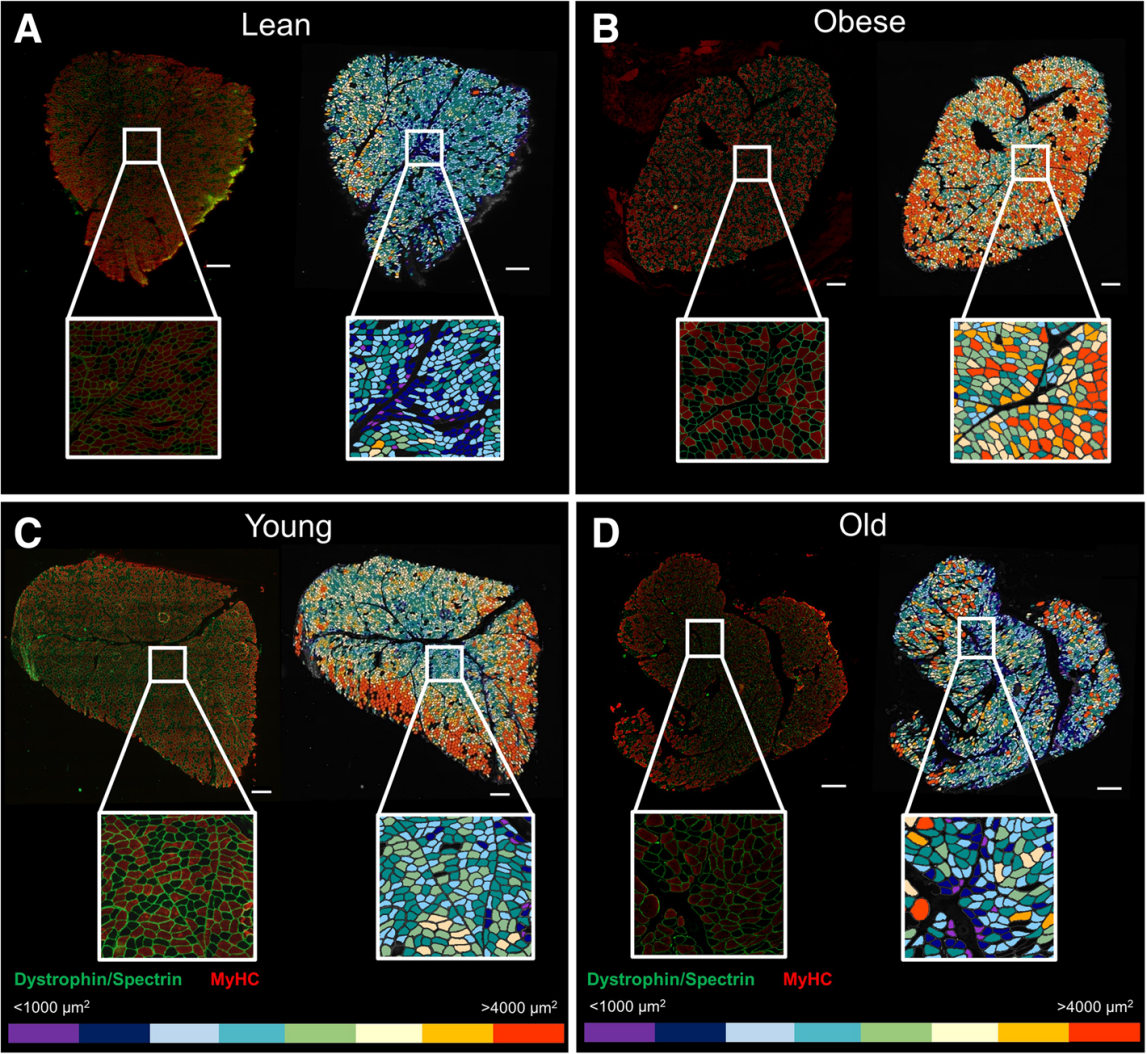
Example of color-coded images as a tool to visually detect changes in myofibers’ size. Color-coded maps were obtained based on the myofibers’ cross-sectional area (from ≤ 1000 to > 4000 μm^2^). Scale bar represents 500 μm. Representative images from **a** lean (male 43 yo, BMI 21.7), **b** obese (male 43 yo, BMI 33.2), **c** young (male 34 yo, BMI 21.71), and **d** old (male 77 yo, BMI 22.5) subjects from the healthy patient cohort. Characteristics of these patients are described in Table 3. Left: original immunofluorescence-stained section. Right: color-coded image. MyHC, myosin heavy chain; yo, years old; BMI, body mass index

Color-coded maps of representative pictures from male lean (43 yo, BMI 21.7, mean CSA 2007 ± 9.6 μm^2^), obese (43 yo, BMI 33.2, mean CSA 3346 ± 16.8 μm^2^), young (34 yo, BMI 21.7, mean CSA 2832 ± 13.5 μm^2^), and old (77 yo, BMI 22.5, mean CSA 2070 ± 14.4 μm^2^) patients analyzed with our macro are shown in Fig. 6, and their morphometry parameters are depicted in Table 3.

**Table 3:** Main characteristics of the patients’ samples depicted Fig. 6

					All fibers		Type I fibers		Type II fibers	
	Gender	Age	BMI (kg/m^2^)	Mean CSA (μm^2^)	Atrophy factor	Hypertrophy factor	Atrophy factor	Hypertrophy factor	Atrophy factor	Hypertrophy factor
Lean	M	43	21.7	2007 ± 9.6	1.5	0.01	2.1	0.02	1.1	0.003
Obese	M	43	33.2	3346 ± 16.8	0.2	0.4	0.2	0.04	0.2	1.1
Young	M	34	21.7	2832 ± 13.5	0.5	0.15	0.7	0.06	0.4	0.23
Old	M	77	22.5	2070 ± 14.4	2.1	0.07	2.3	0.00	1.9	0.12

Deltoid muscle samples from lean, obese, young, and old patients were evaluated using our developed macro in Fiji-ImageJ. Values for CSA are means ± SEM. Atrophy and hypertrophy factors for all, type I, and II myofibers were computed based on a score for the fibers’ CSA size and normalized by the number of each fiber population within a section. *CSA* cross-sectional area. *BMI* body mass index

#### Analysis of diseased human samples using our macro tool in Fiji

**Fig. 7:**
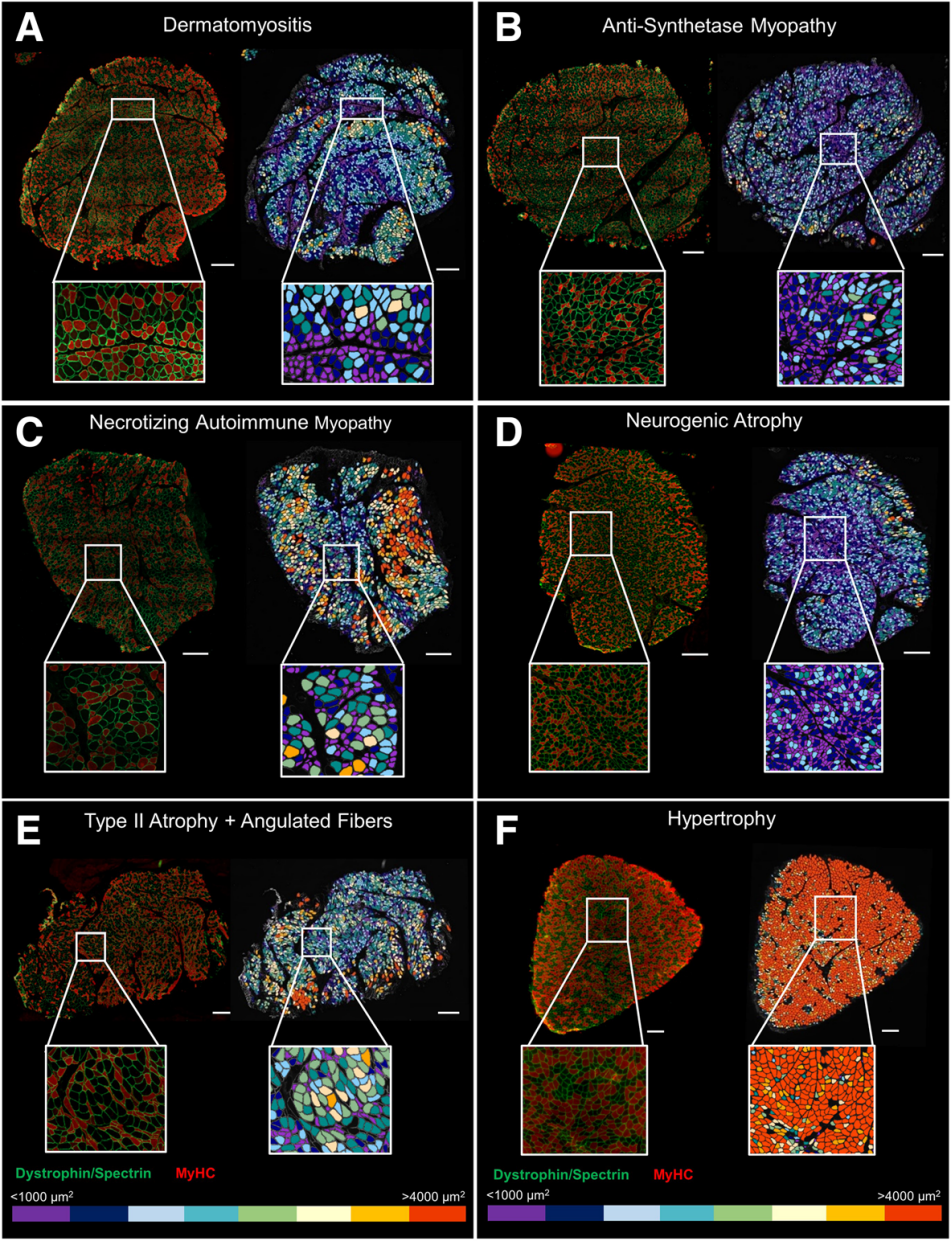
Color-coded maps as a tool to visually detect muscle disease and extreme muscle morphometry. Color-coded maps were obtained based on the myofibers’ cross-sectional area (from ≤ 1000 to > 4000 μm^2^). Scale bar represents 500 μm. Representative images of deltoid muscle from patients diagnosed with **a** dermatomyositis, **b** anti-synthetase myopathy, **c** necrotizing autoimmune myopathy, **d** neurogenic atrophy, and **e** type II atrophy with angulated fibers. **f** Patient showing a large fiber size phenotype. Characteristics of these patients are described in Table 5. Left: original immunofluorescence-stained section. Right: color-coded image. MyHC, myosin heavy chain

We tested our macro under pathological conditions in deltoid muscles from male and female ASM, DM, and NAM patients. We conducted summary statistics of each group of patients (Table 4). The average number of fibers per area was 2086.3 ± 440.8 for ASM, 3175 ± 481 for DM, and 2665.4 ± 675.9 for NAM patients. The mean CSA was 1761.3 ± 495.5, 1410.1 ± 180.6, and 2268.5 ± 365 μm^2^ for ASM, DM, and NAM patients, respectively. We observed that the overall degree of fiber atrophy (as calculated based on the size of the fiber’s CSA) is higher in diseased patients (4.4–6.7) (Table 4) compared to patients with histologically normal muscle (0.2–2.1) (Table 3). In addition, we had access to the samples of two patients with muscle atrophy and a patient whose muscle exhibited extreme fiber morphology (hypertrophy 3.7 vs. < 0.4 in representative healthy patients), and along representative pictures of DM, ASM, and NAM patients, we used these cases to visually highlight the flexibility of the macro (Table 5, Fig. 7).

**Table 4:** Muscle morphometry parameters of patients with pathological muscle

	Muscle pathology		
Morphometry parameters	ASM	DM	NAM
Number of fibers per area^±^	2086.3 ± 440.8	3175.1 ± 481.0	2665.4 ± 675.9
Number of type I (%)	49.5 ± 4.6	53.9 ± 4.2	50.3 ± 5.3
Number of type II (%)	50.5 ± 4.6	46.1 ± 4.2	49.7 ± 5.3
CSA all fibers (μm^2^)	1761.3 ± 495.5	1410.1 ± 180.6	2268.5 ± 365.0
CSA type I (μm^2^)	2022.2 ± 435.0	1569.0 ± 183.7	2722.1 ± 526.9
CSA type II (μm^2^)	1508.4 ± 591.3	1171.6 ± 191.8	1747.4 ± 149.3
Perimeter all fibers (μm)	168.7 ± 25.5	156.8 ± 11.3	196.6 ± 14.1
Perimeter type I (μm)	183.9 ± 19.9	165.1 ± 11.1	216.1 ± 18.6
Perimeter type II (μm)	153.1 ± 32.6	144.4 ± 12.4	174.5 ± 9.3
Major diameter all fibers (μm)	57.3 ± 7.9	54.0 ± 3.7	66.6 ± 5.0
Major diameter type I (μm)	62.2 ± 5.7	56.6 ± 3.5	73.2 ± 6.8
Major diameter type II (μm)	52.1 ± 10.5	49.8 ± 4.1	59.3 ± 3.0
Minor diameter all fibers (μm)	33.7 ± 5.9	30.6 ± 1.9	38.3 ± 3.0
Minor diameter type I (μm)	37.3 ± 4.9	32.9 ± 1.9	43.1 ± 4.4
Minor diameter type II (μm)	30.1 ± 7.3	27.3 ± 2.1	32.7 ± 1.1
Atrophy factor all fibers	6.7 ± 2.5	6.4 ± 1.0	4.4 ± 0.6
Atrophy factor type I	4.6 ± 1.5	5.2 ± 1.0	2.8 ± 0.6
Atrophy factor type II	9.1 ± 3.7	8.2 ± 1.4	6.2 ± 0.8
Hypertrophy factor all	0.09 ± 0.05	0.04 ± 0.03	0.52 ± 0.3
Hypertrophy factor type I	0.10 ± 0.06	0.05 ± 0.03	0.65 ± 0.4
Hypertrophy factor type II	0.10 ± 0.07	0.03 ± 0.03	0.36 ± 0.2

Morphology of total, type I, and type II myofibers of deltoid muscle samples from dermatomyositis (DM), necrotizing autoimmune myopathy (NAM), and anti-synthetase myopathy (ASM) patients evaluated using our macro run in Fiji-ImageJ. Averages of each sample were used for all, type I, and II fiber analysis. Atrophy and hypertrophy factors were computed based on a score for the fibers’ CSA size and normalized by the number of each fiber population within a section. Values are means ± SEM. *CSA* cross-sectional area. ±Normalized number of fibers/area (10 mm^2^)

**Table 5:** Main characteristics of the patients’ samples depicted Fig. 7

					All fibers		Type I fibers		Type II fibers	
	Gender	Age	BMI (kg/m^2^)	Mean CSA (μm^2^)	Atrophy factor	Hypertrophy factor	Atrophy factor	Hypertrophy factor	Atrophy factor	Hypertrophy factor
DM	F	26	NA	1496.6 ± 10.4	5.2	0.002	4.1	0.0004	6.2	0.004
ASM	F	71	NA	1051.4 ± 9.2	9.5	0.004	5.4	0.008	15.0	0.000
NAM	F	62	NA	1938.5 ± 25.1	4.6	0.04	2.8	0.05	7.4	0.02
Neurogenic Atrophy	F	55	17.9	1227.4 ± 8.8	6.5	0.000	4.2	0.000	10.2	0.000
Type II atrophy	M	76	18.3	1782.2 ± 19.5	4.8	0.037	1.1	0.07	8.1	0.002
Hypertrophy	F	60	27.5	4735 ± 35.4	0.3	3.7	0.3	0.6	0.4	7.2

Deltoid muscle samples from dermatomyositis (DM), anti-synthetase myopathy (ASM), necrotizing autoimmune myopathy (NAM), and muscle atrophy patients (neurogenic and type II atrophy), as well as a patient with fiber hypertrophy, were evaluated using our developed macro in Fiji-ImageJ. Values for CSA are means ± SEM. Atrophy and hypertrophy factors for all, type I, and type II myofibers were computed based on a score for the fibers’ CSA size and normalized by the number of each fiber population within a section. *CSA* cross-sectional area, *BMI* mody mass index

We corroborated that our macro still maintains its overall performance, as visually assessed in the segmented output images and color-coded maps, and confirmed our tool applicability across a wide range of human conditions. Furthermore, the area size maps showed their value for the fast visual identification of atrophic/hypertrophic regions (Fig. 7), thus facilitating first impression diagnostics or verifying a diagnostic in clinical settings.

#### Analysis of human pectoral samples using our macro tool in Fiji

**Fig. 8:**
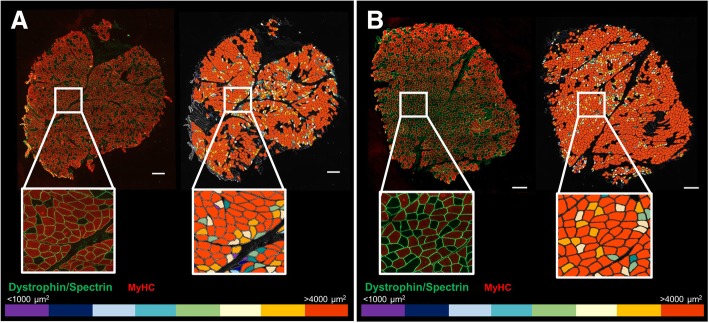
Color-coded images as a tool to detect changes in myofibers’ size in pectoral muscle. Color-coded maps were obtained based on the myofibers’ cross-sectional area (from ≤ 1000 to > 4000 μm^2^). Scale bar represents 500 μm. Examples of images of pectoral muscle biopsies. **a** Male 54 yo, BMI 31.9, and mean CSA 4574.4 ± 43 μm^2^. **b** Male 55 yo, BMI 27.6, and mean CSA 5159.8 ± 42.5 μm^2^. Characteristics of this set of patients are described in Table 6. Left: original immunofluorescence-stained section. Right: color-coded image. MyHC, myosin heavy chain; yo, years old; BMI, body mass index; CSA, cross-sectional area

A convenient feature of our macro tool is the possibility of adjusting exclusion parameters for CSA, minor diameter, and circularity by the user to improve the fiber detection in different physiological (i.e., muscle atrophy in healthy aging) and pathological conditions (muscle disease, obesity). For instance, the pectoral muscle has an overall larger fiber size than deltoid muscle samples. Thus, we adjusted the exclusion parameters before analysis as follows: CSA < 200 and > 20,000 μm^2^, circularity < 0.4, and minor diameter < 8 μm. Our macro was successfully capable of the analysis of pectoral muscle samples, confirming its broad application across different muscle samples (Fig. 8). The average size of a pectoral biopsy was 12.7 ± 1.5 mm^2^, ranging from 2.9 to 18.2 mm^2^. The mean cross-sectional area of all fibers was 4574.8 ± 403.2 μm^2^ (vs. 2399.4 ± 107 μm^2^ in deltoid samples), with an average of 1277.7 ± 234.6 fibers/10mm^2^ area. The morphometry data of these patients for all, type I, and type II fibers are depicted in Table 6.

**Table 6:** Pectoral muscle morphometry parameters

Morphometry parameters	Mean ± SEM
Number of fibers per area^±^	1277.7 ± 234.6
Number of type I (%)	40.1 ± 3.1
Number of type II (%)	59.9 ± 3.1
CSA all fibers (μm^2^)	4574.8 ± 403.2
CSA type I (μm^2^)	4044.8 ± 311.2
CSA type II (μm^2^)	4954.0 ± 542.9
Perimeter all fibers (μm)	296.0 ± 16.2
Perimeter type I (μm)	276.9 ± 13.0
Perimeter type II (μm)	309.6 ± 20.3
Major diameter all fibers (μm)	101.2 ± 5.7
Major diameter type I (μm)	94.9 ± 4.7
Major diameter type II (μm)	105.9 ± 7.1
Minor diameter all fibers (μm)	54.0 ± 2.9
Minor diameter type I (μm)	51.3 ± 2.1
Minor diameter type II (μm)	55.9 ± 3.7
Atrophy factor all fibers	1.2 ± 0.4
Atrophy factor type I	1.4 ± 0.2
Atrophy factor type II	1.1 ± 0.6
Hypertrophy factor all	4.5 ± 0.9
Hypertrophy factor type I	2.7 ± 0.7
Hypertrophy factor type II	5.9 ± 1.6

Morphology of total, type I, and type II myofibers of human pectoral muscle samples evaluated using our macro run in Fiji-ImageJ. Averages of each sample were used for all, type I, and type II fiber analysis. Atrophy and hypertrophy factors were computed based on a score for the fibers’ CSA size and normalized by the number of each fiber population within a section. Values are means ± SEM. *CSA* cross-sectional area. ±Normalized number of fibers/area (10 mm^2^)

## Discussion

### Applicability of the macro analysis and its features across different samples

Here, we presented an accessible, fast, accurate, and reproducible new tool to conduct automatic analyses of human histological samples. Our customized tool runs as a macro in the open-source image processing platform ImageJ® through the Fiji software project. The functionality of the macro was demonstrated in deltoid muscle from young, old, lean, and obese patients, as well as in patients with different pathological conditions and in pectoral muscle, thus confirming that our tool is flexible and broadly applicable to the analyses of a great variety of samples and supporting its value for research purposes as well as in clinical settings. The output color-coded maps based on the fiber’s size proved to be an advantageous feature for the fast and easy visual identification of location-specific atrophy or hypertrophy and a potentially powerful tool for medical diagnosis.

### Method performance

The analysis of muscle morphometry is challenging given the large number of individual fibers within a section. Moreover, different populations of fibers are present in humans, and each of them gives us information about different aspects of the muscle health status [3]. In the past 10 years, an increasing number of software packages for the analysis of muscle images for research purposes has been developed. However, some of these tools require manual image pre-treatment before [13] or user intervention during the analysis [6] resulting in greater evaluation times and prone to bias results.

On the other hand, tools that have demonstrated a more precise segmentation of fibers have either been designed for or tested in rodent muscle [8, 9], they do not run in publicly available platforms [7], or their analysis capacity is limited to a small muscle field rather than the whole muscle [13]. Our program runs in a widely available and free processing package for scientific image analysis. It overall demonstrated a good performance when tested against manual procedures for the detection and identification of fiber populations, with the advantage of performing analyses in the whole muscle, increasing the power of the analyses and reducing partiality in the results. Some of the limitations of our macro include that the program has been set up for the analysis of immunofluorescence-stained slides with the sole identification of type I and II myofibers. We are currently working in setting up the evaluation of fiber subpopulation types (I, IIa, IIx) and hybrid fibers with our macro, but the immunofluorescence-based approach must face the wonted non-specificity of the antibodies (i.e., SC-71 antibody from DSHB, for type IIa fibers, also stains type IIx fibers in humans [14]). Thus, the discrimination of fiber type subpopulations using the automated system has not yet completed. Future developments will also extend the use of the macro for routine immunoperoxidase-stained slides (used in most hospitals).

### The macro demonstrated its functionality in human samples

In cases with normal muscle, we showed that while the number of myofibers and the type I/type II fiber proportion does not change with age or BMI, their morphology is affected by the independent and synergistic actions of these factors. In the analysis of the whole population of fibers, we found that all morphometry parameters were affected by age-by-BMI interactions. The fact that the physiological effects of age and adiposity are intertwined is not surprising. It has been documented that aging results in changes in the distribution of adipose tissue and increased intramuscular lipid accumulation [15], whereas obesity accelerates age-related muscle loss [16].

Moreover, both age [17, 18] and obesity [16, 19, 20] result in decreased muscle strength in humans. In our study, we found significant associations between larger muscle fibers (i.e., CSA, minor diameter) and a greater body mass. A larger muscle cross-sectional area is one of the factors associated with increased fiber force and gain in muscle strength [21]. Therefore, obese subjects that exhibit larger fiber areas would theoretically have higher muscle strength compared to lean. While this had been the case in regard to absolute force values, when strength is adjusted for body mass, the relative muscle strength is, in fact, lower in obese than in lean adults [16, 19]. Thus, in spite of the larger fiber size associated with it, obesity has a negative impact on muscle function.

We also showed that the adaptive responses to age and body mass and their interactions are different between fiber type groups. First, we observed that type II myofibers are more sensitive to aging and that their size significantly decreases (i.e., smaller CSA, perimeter, and major and minor diameters) as age increases. Consistent with these observations, other authors have shown that atrophy of type II fibers and altered fiber shape is a characterizing feature of aging muscle in healthy individuals [2, 4]. Second, although in our population BMI alone did not influence muscle morphometry, we detected significant associations between a greater size (CSA and minor diameter) of type II myofibers and obesity, but these relationships were not significant in the type I population. We also found that the interactions between BMI and age significantly affected all type II, but not all type I parameters. Overall, these data suggest that muscle age- and weight-dependent adaptations might be driven to a greater degree by changes in the type II fiber population. Although in our population we did not observe a slow- to fast-twitching fiber switch (or vice-versa) in response to adiposity variations, we cannot discard changes occurring within the type II fiber subpopulations (i.e., IIa, IIx). In line with this view, a recent study in mice [22] reported the effects of a long-term high-fat diet (12 weeks) in muscle. The diet resulted in muscle lipid infiltration, impairment of the fast-fiber contractile force, and a percentage switch within the fast-twitch fiber type subpopulations, reflected by an increase in type IIa/x fibers, at the expense of decreased type IIb fibers. In this work, we only analyzed type I and type II fiber populations. Hence, the individual effects of BMI on the type II fiber population could be further dissected by characterizing the morphology of the fast fiber type subpopulations and hybrid fibers. Furthermore, our findings suggest that the synergistic effects between obesity and aging on muscle are fiber type specific and could play a role in the development of sarcopenia. However, this point would need to be investigated more thoroughly in future research.

## Conclusions

Overall, we demonstrated that our automated image analysis tool is reliable and suitable for the study of human skeletal muscle research, particularly in the context of the study of sarcopenia correlated with aging and obesity and inflammatory/autoimmune myopathies. Furthermore, the applications of our tool in the medical field are innumerable, from the training of health specialists to the diagnosis of muscle disease providing reproducible and consistent analysis, when the time is of the utmost importance.

## Additional files

Additional file 1: **Figure S1.** Validation of our macro performance against manual analysis. Pearson’s correlation coefficient (*r*) and *p* values are shown for the comparison of our macro vs. manual analysis for the calculation (A) total number of fibers, (B) number of type I and II myofibers, and (C) mean minor diameter of deltoid muscle samples. Mean values of each parameter obtained per sample are plotted. Values with 95% confidence intervals are shown. **Table S1.** Assessment of the effect of gender on deltoid muscle morphometry using our macro in Fiji-ImageJ. **Table S2.** Muscle morphometry parameters of patients with histologically healthy muscle. **Table S3.** Myofibers’ morphology and two-way ANCOVA results. **Table S4.** Comparison of mean myofibers’ morphology across BMI groups. (DOCX 162 kb)

Additional file 2: Macro script used for the analysis of muscle morphometry. (TXT 10 kb)

Additional file 3: Tutorial for the quantification of muscle fiber morphometry using the macroIMRB. (DOCX 2541 kb)

## Abbreviations

ANCOVA: Analysis of covariance
ASM: Anti-synthetase myopathy
BMI: Body mass index
BW: Body weight
C1/C2: Channel 1, channel 2
CSA: Cross-sectional area
DM: Dermatomyositis
MyHC: Myosin heavy chain
NAM: Necrotizing autoimmune myopathy
PBS: Phosphate buffer saline
SAS: State-of-the-art statistical software
SEM: Standard error of the mean
TIF: Tagged image format
xls: Excel spreadsheet
yo: Years old

## References

[1] Pant I, Chaturvedi S, Bala K, Kushwaha S. Muscle histopathology in today’s era of molecular genetics: role and limitations. Ann Indian Acad Neurol. 2015;18(4):398. 10.4103/0972-2327.165455 PMC4683876 26713009

[2] Nilwik Rachel, Snijders Tim, Leenders Marika, Groen Bart B.L., van Kranenburg Janneau, Verdijk Lex B., van Loon Luc J.C. (2013). The decline in skeletal muscle mass with aging is mainly attributed to a reduction in type II muscle fiber size. Experimental Gerontology.

[3] Schiaffino Stefano, Reggiani Carlo (2011). Fiber Types in Mammalian Skeletal Muscles. Physiological Reviews.

[4] Wang Yichen, Pessin Jeffrey E. (2013). Mechanisms for fiber-type specificity of skeletal muscle atrophy. Current Opinion in Clinical Nutrition and Metabolic Care.

[5] Kostrominova TY, Reiner DS, Haas RH, Ingermanson R, McDonough PM (2013). Automated methods for the analysis of skeletal muscle fiber size and metabolic type. Int RevCell Mol Biol.

[6] Smith LR, Barton ER (2014). SMASH–semi-automatic muscle analysis using segmentation of histology: a MATLAB application. Skelet Muscle.

[7] Pertl C, Eblenkamp M, Pertl A, Pfeifer S, Wintermantel E, Lochmüller H, et al. A new web-based method for automated analysis of muscle histology. BMC Musculoskelet Disord. 2013;14(1):26. 10.1186/1471-2474-14-26 PMC3560198 23324401

[8] Mayeuf-Louchart A, Hardy D, Thorel Q, Roux P, Gueniot L, Briand D, et al. MuscleJ: a high-content analysis method to study skeletal muscle with a new Fiji tool. Skelet Muscle. 2018;8(1):25. 10.1186/s13395-018-0171-0 PMC6091189 30081940

[9] Bergmeister Konstantin D., Gröger Marion, Aman Martin, Willensdorfer Anna, Manzano-Szalai Krisztina, Salminger Stefan, Aszmann Oskar C. (2016). Automated muscle fiber type population analysis with ImageJ of whole rat muscles using rapid myosin heavy chain immunohistochemistry. Muscle & Nerve.

[10] De Bleecker JL, Lundberg IE, de Visser M (2013). 193rd ENMC international workshop pathology diagnosis of idiopathic inflammatory myopathies 30 November–2 December 2012, Naarden, Netherlands. Neuromuscular Disorders.

[11] Dubowitz V. and Sewry C. Definition of pathological changes seen in muscle biopsies. In: Houston MJ, Cook L, editors. Muscle Biopsy A Practical Approach. 3rd ed. Saunders Ltd; 2007. p. 91–2.

[12] Schindelin J, Arganda-Carreras I, Frise E, Kaynig V, Longair M, Pietzsch T (2012). Fiji: an open-source platform for biological-image analysis. Nat Methods.

[13] Wen Y, Murach KA, Vechetti IJ, Fry CS, Vickery C, Peterson CA (2017). MyoVision: software for automated high-content analysis of skeletal muscle immunohistochemistry. J Appl Physiol.

[14] Bloemberg D, Quadrilatero J (2012). Rapid determination of myosin heavy chain expression in rat, mouse, and human skeletal muscle using multicolor immunofluorescence analysis. PLoS One.

[15] Kuk JL, Saunders TJ, Davidson LE, Ross R (2009). Age-related changes in total and regional fat distribution. Ageing Res Rev.

[16] Tomlinson David J., Erskine Robert M., Winwood Keith, Morse Christopher Ian, Onambélé Gladys L. (2014). Obesity decreases both whole muscle and fascicle strength in young females but only exacerbates the aging-related whole muscle level asthenia. Physiological Reports.

[17] Larsson L, Grimby G, Karlsson J (1979). Muscle strength and speed of movement in relation to age and muscle morphology. J Appl Physiol.

[18] Lindle R, Metter E, Lynch N, Fleg J, Fozard J, Tobin J (1997). Age and gender comparisons of muscle strength in 654 women and men aged 20–93 yr. J Appl Physiol.

[19] Maffiuletti NA, Jubeau M, Munzinger U, Bizzini M, Agosti F, De Col A (2007). Differences in quadriceps muscle strength and fatigue between lean and obese subjects. Eur J Appl Physiol.

[20] Blimkie CJ, Sale DG, Bar-Or O (1990). Voluntary strength, evoked twitch contractile properties and motor unit activation of knee extensors in obese and non-obese adolescent males. Eur J Appl Physiol Occup Physiol.

[21] Miller AEJ, MacDougall J, Tarnopolsky M, Sale D (1993). Gender differences in strength and muscle fiber characteristics. Eur J Appl Physiol Occup Physiol.

[22] Eshima H, Tamura Y, Kakehi S, Kurebayashi N, Murayama T, Nakamura K (2017). Long-term, but not short-term high-fat diet induces fiber composition changes and impaired contractile force in mouse fast-twitch skeletal muscle. Physiol Rep.

