# Supplementary material for: Automated image-analysis method for the quantification of fiber morphometry and fiber type population in human skeletal muscle
Source: Skelet Muscle. 2019 May 27;9:15. doi: 10.1186/s13395-019-0200-7 (PMC6537183; doi:10.1186/s13395-019-0200-7)
Supplement: Supplementary file 1 — Figure S1. Validation of our macro performance against manual analysis. Pearson’s correlation coefficient (r) and p values are shown for the comparison of our macro vs. manual analysis for the calculation (A) total number of fibers, (B) number of type I and II myofibers, and (C) mean minor diameter of deltoid muscle samples. Mean values of each parameter obtained per sample are plotted. Values with 95% confidence intervals are shown. Table S1. Assessment of the effect of gender on deltoid muscle morphometry using our macro in Fiji-ImageJ. Table S2. Muscle morphometry parameters of patients with histologically healthy muscle. Table S3. Myofibers’ morphology and two-way ANCOVA results. Table S4. Comparison of mean myofibers’ morphology across BMI groups. (DOCX 162 kb) [file 13395_2019_200_MOESM1_ESM.docx]

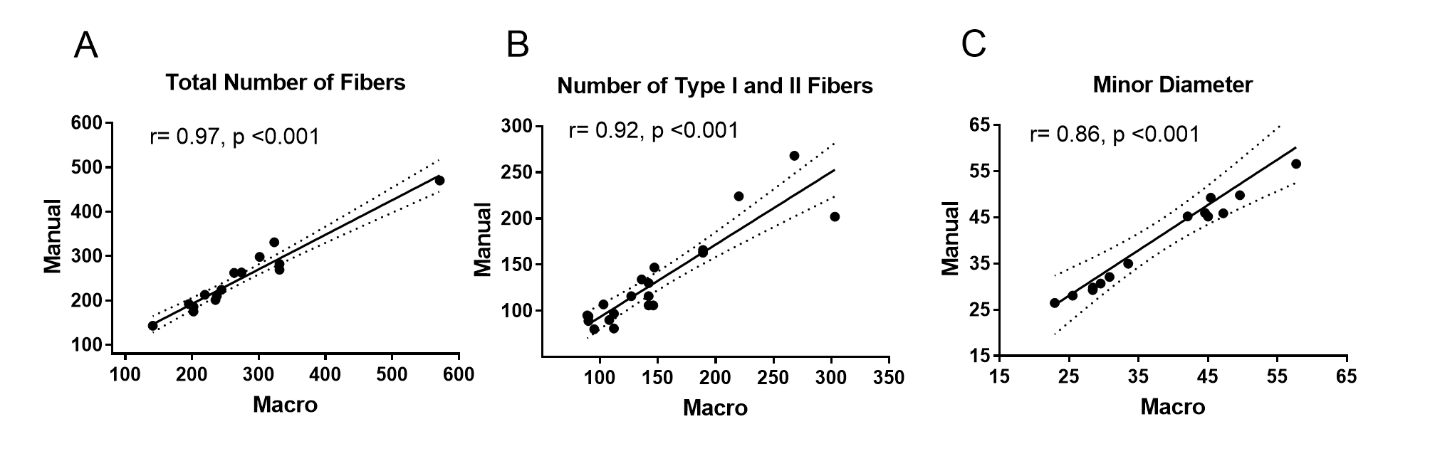


**Figure S1.** *Validation of our Macro performance against Manual Analysis.* Pearson’s correlation coefficient (r) and p values are shown for the comparison of our macro vs. manual analysis for the calculation (A) total number of fibers, (B) number of type I and II myofibers, and (C) mean minor diameter of deltoid muscle samples. Mean values of each parameter obtained per sample are plotted. Values with 95% confidence intervals are shown.

**Assessment of the effect of gender on myofibers’ morphometry**

**Methods**

To assess the potential covariate effects between sex and fiber morphometry we conducted linear Pearson’s correlation analysis between fiber size [CSA, perimeter, major and minor diameter (all fibers)] and fiber number against gender. We gave values to males = 0 and females = 1.

We also carried out separate statistical analysis to assess the main effect of gender on muscle fiber morphometry features independently of BMI and Age (i.e., one-way ANCOVA with age and BMI as covariates in the model). Tukey-Kramer *post hoc* test was used to determine differences between groups. Statistical analyses were performed using SAS Enterprise Guide OnDemand (SAS Institute, Inc., Cary, NC) and significance was set at p <0.05.

**Results**

We observed that fiber size (CSA r=-0.45, p=0.0004; Perimeter r=-0.47, p=0.0003; Major diameter r=-0.49, p=0.0001; Minor diameter r=-0.42, p=0.001, vs. gender) and fiber number (r=0.28, p=0.034 vs. gender) were significantly correlated with gender. These data suggest that males have larger fibers, hence a lesser number of fibers/area than females. These results were confirmed by our one-way ANCOVA analysis, showing that muscle fibers in males have significantly larger CSA, perimeter, and major and minor diameters, but a smaller number of fibers per area compared to females (Suppl. Table 1). Thus, our analyses were able to distinguish clear differences between males and females in fiber size and number. However, given the existence of sex-related differences affecting the morphometry of muscle fibers in our population, we corrected the potential confounding effect of gender by conducting an analysis of covariance (i.e., two-way ANCOVA with gender as a covariate in the model). Thereby increasing the power to detect an effect of other variants (i.e., age, BMI) directly influencing muscle morphometry.

**Table S1.**

| **Morphometry Parameter** |  | **Males** | | |  | **Females** | | |  | **One-way ANCOVA** |
| --- | --- | --- | --- | --- | --- | --- | --- | --- | --- | --- |
| Number of fibers per area^±^ |  | 2023.1 | ± | 148.3^a^ |  | 2517.8 | ± | 140.7^b^ |  | 0.02 |
| CSA (µm^2^) |  | 2799.9 | ± | 130.7^b^ |  | 2039.0 | ± | 124^a^ |  | <.0001 |
| Perimeter (µm) |  | 225.8 | ± | 6.1^b^ |  | 189.4 | ± | 5.8^a^ |  | <.0001 |
| Major Diameter (µm) |  | 77.3 | ± | 2.1^b^ |  | 64.3 | ± | 2^a^ |  | <.0001 |
| Minor Diameter (µm) |  | 44.3 | ± | 1.2^b^ |  | 37.7 | ± | 1.1^a^ |  | 0.0002 |

Deltoid muscle samples were evaluated using our macro in Fiji-ImageJ. One-Way ANCOVA (age and BMI as covariates in the model) results for gender effect on fiber number and size. ^±^Normalized number of fibers/area (10 mm^2^). Values are LSmeans ± SEM. Means with different letter superscripts are statistically different (Tukey-Kramer test, p<0.05).

**Table S2. Muscle morphometry parameters of patients with histologically healthy muscle**

| **Myofibers’ morphology** | **Mean ± SEM** |
| --- | --- |
| Number of fibers per area^±^ | 2283.47 ± 109.5 |
| Number of type I (%) | 47.4 ± 1.7 |
| Number of type II (%) | 52.6 ± 1.7 |
| CSA all fibers (µm^2^) | 2399.4 ± 106.5 |
| CSA Type I (µm^2^) | 2332.9 ± 87.9 |
| CSA type II (µm^2^) | 2446.8 ± 138.2 |
| Perimeter all fibers (µm) | 206.6 ± 5 |
| Perimeter type I (µm) | 202.9 ± 3.8 |
| Perimeter type II (µm) | 210.2 ± 6.4 |
| Major Diameter all fibers (µm) | 70.5 ± 1.7 |
| Major Diameter type I (µm) | 69.1 ± 1.3 |
| Major Diameter type II (µm) | 71.7 ± 2.2 |
| Minor Diameter all fibers (µm) | 40.9 ± 1 |
| Minor Diameter type I (µm) | 41.2 ± 0.8 |
| Minor Diameter type II (µm) | 40.5 ± 1.3 |

Morphology of total, type I and type II myofibers of human deltoid muscle samples from patients with histologically healthy muscle were evaluated using our macro run in Fiji-ImageJ. ^±^Normalized number of fibers/area (10 mm^2^). Averages of each sample were used for all, type I, and II fiber analysis. Values are means ±SEM. CSA: cross-sectional area.

**Table S3. Myofibers’ morphology, Two-way ANCOVA results**

| **Myofibers’ morphology** | **Age** | **BMI** | **BMI x Age** |
| --- | --- | --- | --- |
| Number of fibers per area^±^ | 0.72 | **0.07** | 0.13 |
| Number of type I (%) | 0.47 | 0.98 | 0.52 |
| Number of type II (%) | 0.47 | 0.98 | 0.52 |
| CSA all fibers (µm^2^) | **0.045** | 0.13 | **0.007** |
| CSA Type I (µm^2^) | 0.94 | 0.53 | 0.11 |
| CSA type II (µm^2^) | **0.0008** | 0.11 | **0.017** |
| Perimeter all fibers (µm) | **0.03** | 0.21 | **0.005** |
| Perimeter type I (µm) | 0.94 | 0.57 | **0.08** |
| Perimeter type II (µm) | **0.0009** | 0.28 | **0.017** |
| Major Diameter all fibers (µm) | **0.02** | 0.18 | **0.002** |
| Major Diameter type I (µm) | 0.84 | 0.52 | **0.03** |
| Major Diameter type II (µm) | **0.0008** | 0.23 | **0.008** |
| Minor Diameter all fibers (µm) | **0.0003** | 0.12 | **0.002** |
| Minor Diameter type I (µm) | 0.28 | 0.53 | **0.05** |
| Minor Diameter type II (µm) | **<0.0001** | 0.12 | **0.004** |

Morphology of all, type I and type II myofibers of deltoid muscle samples was evaluated using our developed macro in Fiji-ImageJ. ^±^Normalized number of fibers/area (10 mm^2^). Gender-adjusted analysis of covariance (2-way ANCOVA), p-values for main effects of Age, BMI and their interactions are shown. In red p<0.05, in green p<0.1.

**Table S4**. **Comparison of mean myofibers’ morphology across BMI groups**

|  | **BMI group (kg/m^2^)** | | | | | | | | | | | |  |
| --- | --- | --- | --- | --- | --- | --- | --- | --- | --- | --- | --- | --- | --- |
| **Myofibers’ morphology** | **17-18.5** | | | **18.5 -24.9** | | | **25-29.9** | | | **>30** | | | |
| Number of fibers per area^±^ | 2793.8 | ± | 313.2 | 2361.5 | ± | 156.9 | 2484.8 | ± | 249.7 | 1627.6 | ± | 316.7 | |
| Number of type I (%) | 48.4 | ± | 5.4 | 46.9 | ± | 2.7 | 45.9 | ± | 4.3 | 45.9 | ± | 5.5 | |
| Number of type II (%) | 51.6 | ± | 5.4 | 53.1 | ± | 2.7 | 54.1 | ± | 4.3 | 54.1 | ± | 5.5 | |
| CSA all fibers (µm^2^) | 2071.8 | ± | 240.4 | 2297.4 | ± | 120.4 | 2152.8 | ± | 191.6 | 2812.5 | ± | 243.1 | |
| CSA Type I (µm^2^) | 2185.7 | ± | 249.5 | 2327.7 | ± | 125 | 2157.8 | ± | 199 | 2614.4 | ± | 252.3 | |
| CSA type II (µm^2^) | 1951.8 | ± | 296.8 | 2190.7 | ± | 148.6 | 2164.5 | ± | 236.6 | 2921.5 | ± | 300.1 | |
| Perimeter all fibers (µm) | 187.2 | ± | 11.1 | 203.5 | ± | 5.5 | 198.2 | ± | 8.8 | 220.1 | ± | 11.2 | |
| Perimeter type I (µm) | 193.2 | ± | 10.8 | 202.4 | ± | 5.4 | 198.0 | ± | 8.6 | 213.6 | ± | 10.9 | |
| Perimeter type II (µm) | 183.3 | ± | 14.2 | 202.1 | ± | 7.1 | 199.0 | ± | 11.3 | 222.9 | ± | 14.3 | |
| Major Diameter all fibers (µm) | 63.8 | ± | 3.6 | 69.5 | ± | 1.8 | 67.6 | ± | 2.9 | 75.1 | ± | 3.7 | |
| Major Diameter type I (µm) | 65.6 | ± | 3.5 | 69.0 | ± | 1.8 | 67.3 | ± | 2.8 | 72.6 | ± | 3.6 | |
| Major Diameter type II (µm) | 62.6 | ± | 4.6 | 69.1 | ± | 2.3 | 68.0 | ± | 3.7 | 76.3 | ± | 4.7 | |
| Minor Diameter all fibers (µm) | 37.0 | ± | 2 | 40.1 | ± | 1 | 38.7 | ± | 1.6 | 43.7 | ± | 2 | |
| Minor Diameter type I (µm) | 39.6 | ± | 2.1 | 41.1 | ± | 1.1 | 39.7 | ± | 1.7 | 43.4 | ± | 2.1 | |
| Minor Diameter type II (µm) | 34.9 | ± | 2.4 | 38.5 | ± | 1.2 | 38.0 | ± | 1.9 | 43.3 | ± | 2.5 | |

Morphology of all, type I and type II myofibers of deltoid muscle samples was evaluated using our developed macro in Fiji-ImageJ. Analysis of covariance (2-way ANCOVA) results by BMI-group are shown. Averages of each sample were used for all, type I, and II fiber analysis. ^±^Normalized number of fibers/area (10 mm^2^). Values are LSmeans ± SEM. For each parameter, means in the same row that have different superscripts are significantly different from each other (Tukey-Kramer test, p<0.05). CSA: cross-sectional area. BMI: Body mass index.
