# Supplementary material for: Automated image-analysis method for the quantification of fiber morphometry and fiber type population in human skeletal muscle
Source: Skelet Muscle. 2019 May 27;9:15. doi: 10.1186/s13395-019-0200-7 (PMC6537183; doi:10.1186/s13395-019-0200-7)
Supplement: Supplementary file 3 — Tutorial for the quantification of muscle fiber morphometry using the macroIMRB. (DOCX 2541 kb) [file 13395_2019_200_MOESM3_ESM.docx]

***Image Acquisition and image format***

- Microscope: Zeiss Axio
- Objective: 10X objective
- Channel 1: Membrane, FICT 488 (green)
- Channel 2: MyHC, Cy549 (red)
- Format: from the Axio microscope, export the images of the individual channels in a TIF format without compression (16 bit).
  - In the Axio 🡪 Click on the Processing tab

Method 🡪 Image Export

- - - File type: Tagged Image File Format (tiff)
    - 8bit - deselect
    - **Compression: NONE**
    - **Original Data - select**
    - **Apply Display Curve and Channel Color - deselect**
    - Export to: choose file where all images are
    - Create folder – select
- You will have an ORG file for each channel. These file names are recognized by the macroIMRB: FileNameNoSpaces_C1_ORG, and FileNameNoSpaces_C2_ORG

***Automated Morphometric Analyses using the MacroIMRB***

The macro can run in Fiji in a computer with available Java 8 runtime. The image processing is compatible with Windows 64-bit (XP, Vista, 7, 8 and 10), Mac OS (X 10.8), Mac OS X and Linux (64 and x86 architectures).

1. Download the macro script (text file) and save it into a suitable folder.
2. Install and open FIJI- ImageJ (https://fiji.sc/)
3. Go to Plugins🡪 macros 🡪 run 🡪 and click on the macro file
4. It is possible to modify the following variables for detection in a pop-up window that will appear before running the analysis. These are the default parameters:


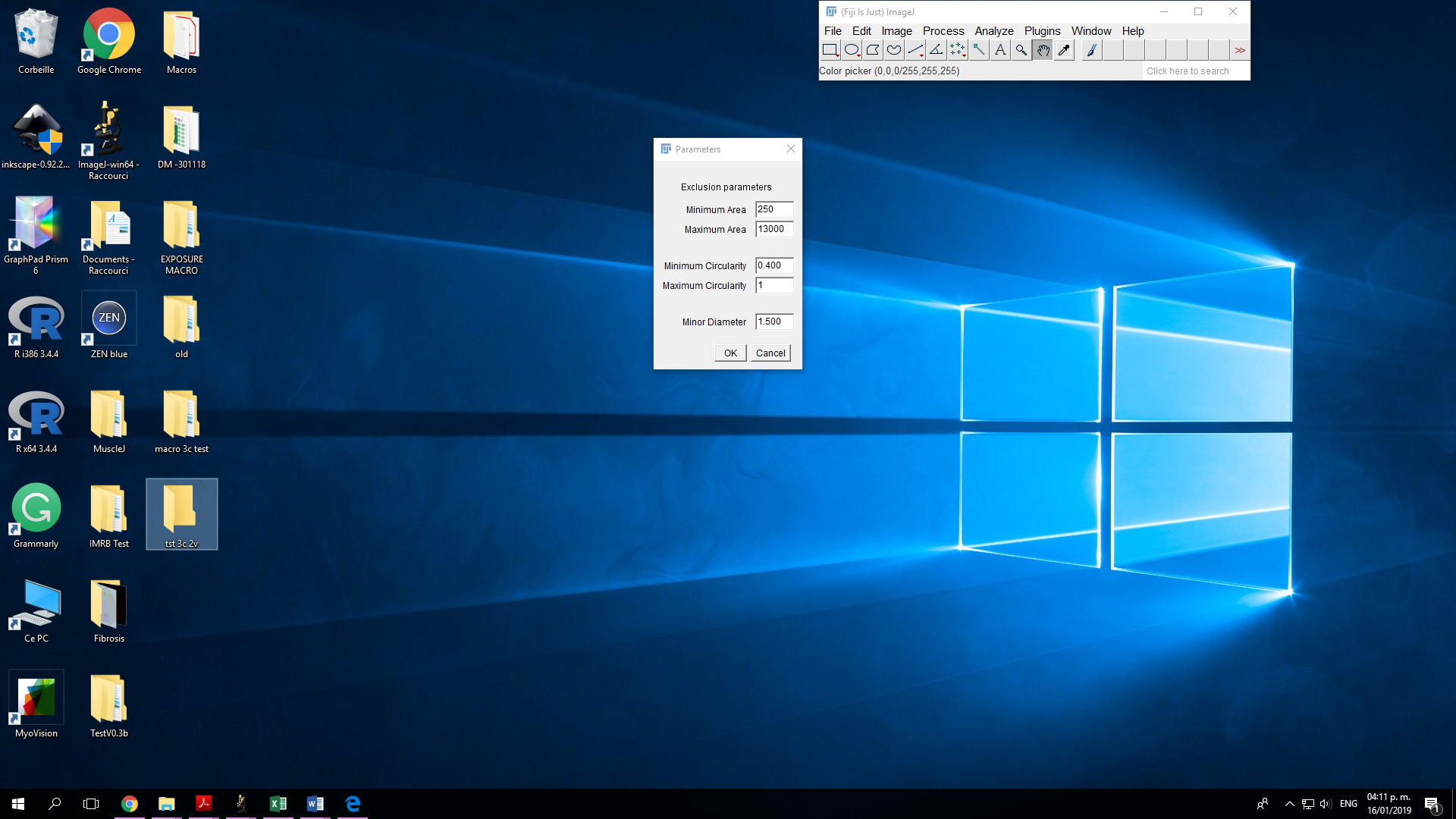


1. A window will open: choose the folder were the images to analyze (with the appropriate format for the macroIMRB) are saved. Images can be saved in subfolders within the main folder.
2. The macro will run automatically and will analyze all the images contained in the main folder (including those in subfolders).
3. The morphometry parameters: CSA (µm^2^), perimeter (µm), major (µm) and minor diameters (µm), and circularity (0-1) will be computed for all myofibers. The results obtained will be automatically saved as excel (.xls) files for all, type I, and type II myofibers separately.


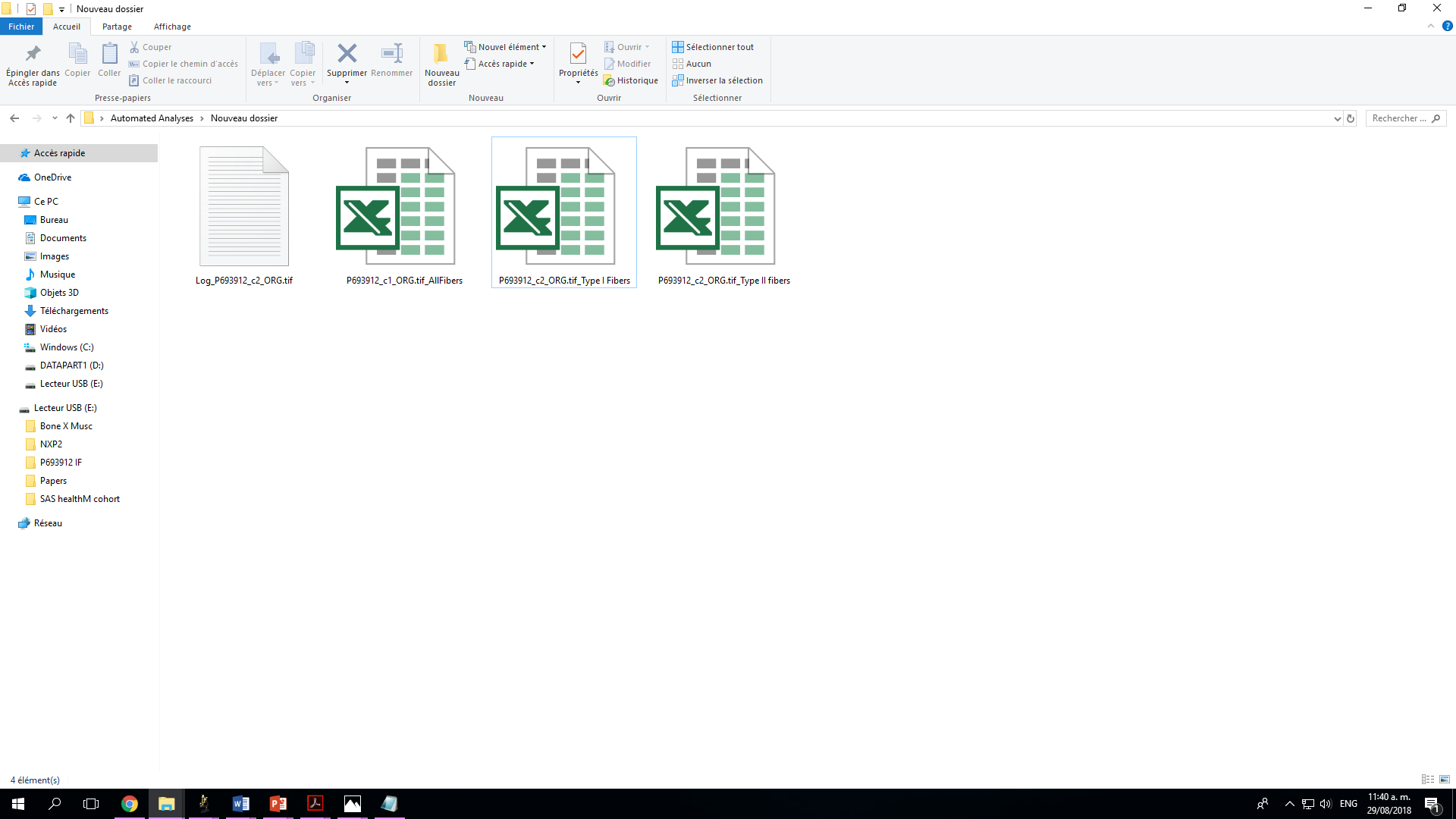


1. To facilitate visual analysis of fiber size distribution (area and minor diameter), a color-coded map for each section will be created (these parameters can be modified by the user directly in the macro script – default parameters are set up for adults).

**Table 1.** Color key for Area and minor diameter size maps

|  | Cross-Sectional Area (µm^2^) | | |  | Minor Diameter (µm) |
| --- | --- | --- | --- | --- | --- |
|  | Adults |  | Children |  | Adults/Children |
| Dark orchid | ≤1000 |  | ≤300 |  | ≤10 |
| Night Blue | > 1000 to ≤1500 |  | > 300 to ≤ 600 |  | > 10 to ≤ 20 |
| Cyan blue | >1500 to ≤ 2000 |  | > 600 to ≤ 1000 |  | >20 to ≤ 30 |
| Dark Cyan | >2000 to ≤ 2500 |  | >1000 to ≤ 1300 |  | >30 to ≤ 40 |
| Dark Sea Green | > 2500 to ≤ 3000 |  | > 1300 to ≤ 1600 |  | > 40 to ≤ 50 |
| Yellow | > 3000 to ≤ 3500 |  | > 1600 to ≤ 2000 |  | > 50 to ≤ 60 |
| Orange | >3500 to ≤ 4000 |  | >2000 to ≤ 3000 |  | >60 to ≤ 70 |
| Red | >4000 |  | >3000 |  | >70 |


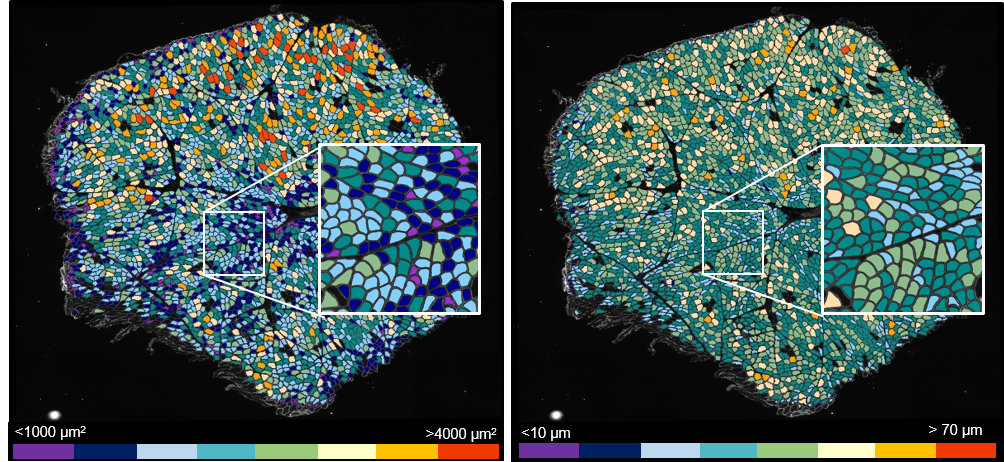


1. A log file for each sample with the total area of tissue detected (µm^2^), and the number of total, type I, and type fibers quantified will be saved in the same folder selected for analysis.


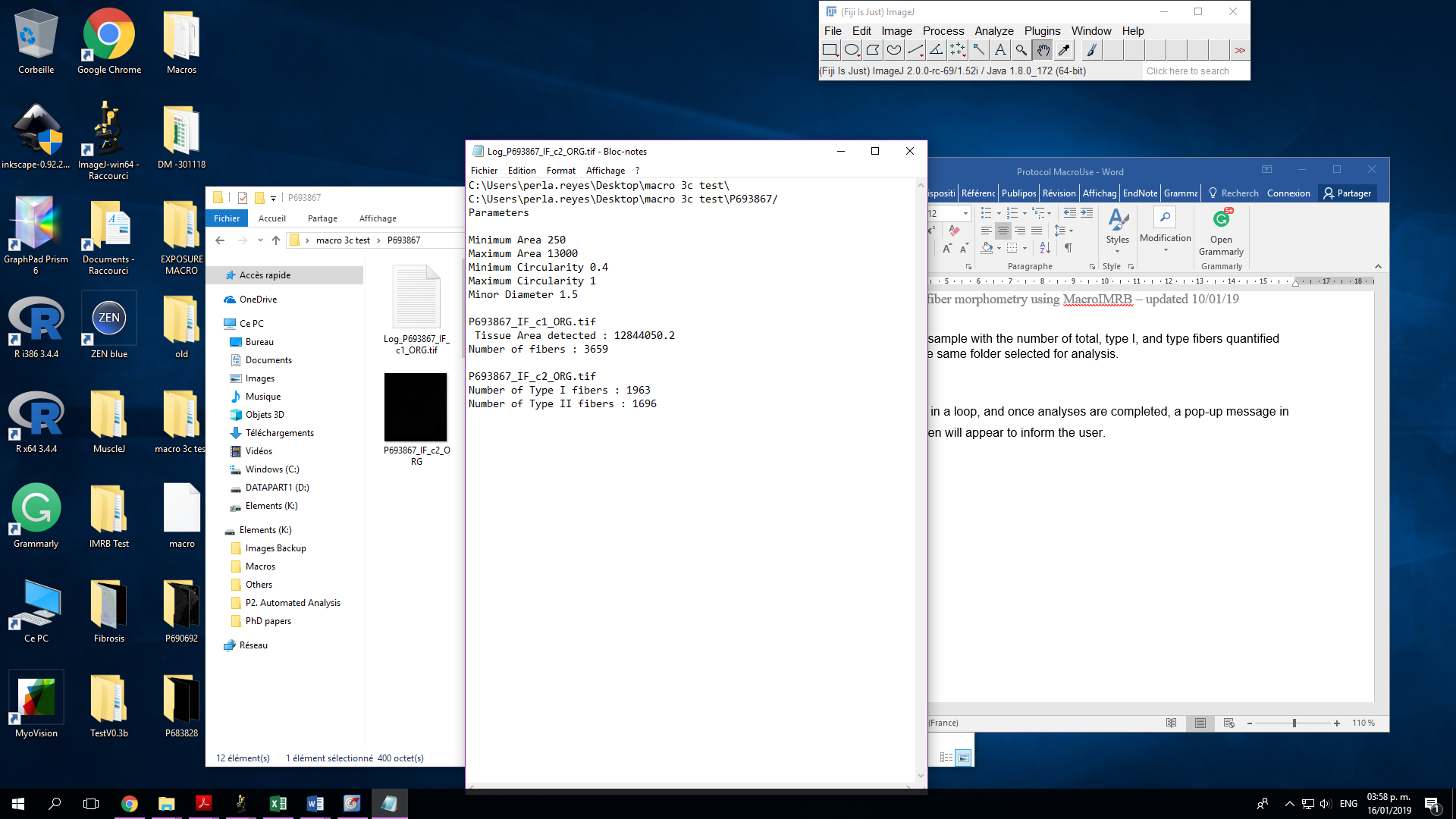


1. The program runs in a loop, and once analyses are completed, a pop-up message in the computer screen will appear to inform the user.
